# Past, Present, and Future of Naturally Occurring Antimicrobials Related to Snake Venoms

**DOI:** 10.3390/ani13040744

**Published:** 2023-02-19

**Authors:** Nancy Oguiura, Leonardo Sanches, Priscila V. Duarte, Marcos A. Sulca-López, Maria Terêsa Machini

**Affiliations:** 1 Laboratory of Ecology and Evolution, Instituto Butantan, Av. Dr. Vital Brasil 1500, São Paulo 05503-900, Brazil; 2 Aquatic Microbiology and Technological Applications, Department of Microbiology and Parasitology, Faculty of Biological Sciences, Universidad Nacional Mayor de San Marcos, Av. Venezuela Cdra. 34 s/n, Ciudad Universitaria, Lima 15081, Peru; 3 Laboratory of Peptide Chemistry, Department of Biochemistry, Institute of Chemistry, University of São Paulo, Av. Prof. Lineu Prestes, 748, São Paulo 05508-000, Brazil

**Keywords:** snake venoms, antimicrobial activity, snake toxins, snake immunity, rattlesnakes, cathelicidins, defensins, genes, peptides

## Abstract

**Simple Summary:**

A critical global health problem is microbial resistance to antibiotics. In order to further discuss this issue and search for practical means to overcome such problems, we reviewed the bibliography related to snake venoms, their proteins, and peptides with antimicrobial activity because many of them have the potential to become alternative antimicrobial agents or serve as lead compounds for the development of new ones. Among the proteins classified according to their structures are lectins, metalloproteinases, L-amino acid oxidases, phospholipases type A_2_, cysteine-rich secretory proteins, and serine proteinases. Among the oligopeptides are waprins, cardiotoxins, cathelicidins, and β-defensins. The list includes natural and synthetic small peptides, many derived from the proteins and the oligopeptides cited above. In vitro, all these snake-venom components are active against bacteria, fungi, parasites, and/or viruses pathogenic to humans. Some have also been tested in laboratory animals. In addition to organizing and discussing such an expressive amount of information, we propose here a multidisciplinary approach that includes sequence phylogeny as a way to better understand the relationship between amino-acid sequence and antimicrobial activity.

**Abstract:**

This review focuses on proteins and peptides with antimicrobial activity because these biopolymers can be useful in the fight against infectious diseases and to overcome the critical problem of microbial resistance to antibiotics. In fact, snakes show the highest diversification among reptiles, surviving in various environments; their innate immunity is similar to mammals and the response of their plasma to bacteria and fungi has been explored mainly in ecological studies. Snake venoms are a rich source of components that have a variety of biological functions. Among them are proteins like lectins, metalloproteinases, serine proteinases, L-amino acid oxidases, phospholipases type A_2_, cysteine-rich secretory proteins, as well as many oligopeptides, such as waprins, cardiotoxins, cathelicidins, and β-defensins. In vitro, these biomolecules were shown to be active against bacteria, fungi, parasites, and viruses that are pathogenic to humans. Not only cathelicidins, but all other proteins and oligopeptides from snake venom have been proteolyzed to provide short antimicrobial peptides, or for use as templates for developing a variety of short unnatural sequences based on their structures. In addition to organizing and discussing an expressive amount of information, this review also describes new β-defensin sequences of *Sistrurus miliarius* that can lead to novel peptide-based antimicrobial agents, using a multidisciplinary approach that includes sequence phylogeny.

## 1. Introduction

Animals and plants possess an arsenal of potent macromolecules to protect themselves against infections. Such an arsenal is chemically heterogeneous and includes proteins and peptides with antimicrobial activity [1,2].

In the animal kingdom, reptiles are organisms of great adaptability, a feature that allows them to survive in several environments or ecological niches. Therefore, reptiles have undergone significant diversification and have been considered intermediates between ectothermic anamniotes (fish and amphibians) and endothermic amniotic animals (birds and mammals) [3]. Hence, snakes are widely distributed throughout the world [4].

Snake venoms are mixtures of a variety of pharmacologically active chemicals, under study mainly for scientific and medical interest. Many of the published studies focusing these natural sources aim at disclosing the biological activities of toxins or developing new molecules with high therapeutic indexes [5]. Furthermore, expanding the knowledge of snake immunity can be quite useful in the battle against pathogenic microorganisms that are resistant to antibiotics [6]. Indeed, bacterial antimicrobial resistance (AMR) has emerged as one of the leading public health threats of the 21st century, so every year the World Health Organization (WHO) organizes the global campaign, World Antimicrobial Awareness Week (WAAW), aiming to improve awareness and understanding of AMRs as well as to encourage good practices for treating bacterial infections. The theme of WAAW 2022 was “Preventing Antimicrobial Resistance Together”.

In view of such relevant information and aiming to contribute to the elucidation of snakes’ abilities to survive in different ecological niches, we concluded that it would be particularly interesting to shed light on topics related to snakes’ defense against microorganisms. Thus, this review organizes and discusses part of the existing knowledge of snake immunity, snake-venom toxins, and antimicrobial proteins and peptides (AMPs), or host defense peptides (HDPs) found in snake venoms. It is worth stressing here that last June, a Brazilian research group tracked and published the scientific production of our country related to peptides from snake venoms [7], confirming that Brazilian research in this field is strong. Indeed, our pioneering studies mostly focused on accidents and treatments, then on biological activities of toxins and, in the 21st century on new functions, such as anti-inflammatory, antitumor, analgesic, and antimicrobial activities [7].

In comparison with conventional antibiotics, AMPs inhibit the growth of, and/or rapidly kill, pathogenic microorganisms with higher efficiency, because they mainly target bacterial and fungal cell membranes [8,9]. In addition, the most significant advantage of these biopolymers over antibiotics is the fact that they do not induce the generation of resistant mutant microorganisms after sequential exposure at concentrations close to their minimum inhibitory concentrations (MICs) [10,11]. Although all AMPs known so far are catalogued in APD3 (https://aps.unmc.edu/, accessed on 7 January 2023), a database that also includes AMPs related to snake venoms or components of this natural source, it is difficult to order them in terms of potency, because the MICs reported were determined using different experimental approaches (like radial diffusion or standard disc diffusion assay, Bactec TB-460 radiometric method [12], determination of MICs in liquid media using optical density or colony-forming units) or tests with a fixed concentration of AMP. Even so, it is feasible to trace a path to use these biomolecules as candidates for therapeutic drugs, or as lead compounds for the development of novel antimicrobial agents.

## 2. The Immunity of Snakes

Reptiles are ectothermic animals, since they are not able to control their internal temperature, requiring strong seasonal shifts in behavior to maintain the body temperature [13]. Like mammals, reptile immunity is complex and comprises innate and adaptive immune systems, including cell-mediated and humoral responses [13]. So, this is an interesting group to be studied regarding host defense, since the innate immune system of reptiles—which includes nonspecific leukocytes, antimicrobial peptides, and the complement system—responds vigorously and quickly, allowing these animals to combat a wide range of pathogens and thrive in numerous environments. Such broad feedback is typically followed by a moderate adaptive immune response [14]. Since relatively little is known about it, and even less in snakes, this revision will focus on naturally occurring antimicrobial proteins, oligopeptides, and short peptides (AMPs) found in snake venoms.

Like lizards and amphisbaenians, snakes belong to the order Squamata. These reptiles are distributed throughout almost every environment of the globe, except for the polar caps. There are aquatic and terrestrial snakes. Thus in our planet’s environments, these animals occupy fossorial, terrestrial, and arboreal niches; they live in forests, savannas, or deserts; while some are venomous, others are not [4].

According to Grego et al., 2006 [15] the cells commonly found in snake blood are erythrocytes, thrombocytes, and leukocytes. Among the last are lymphocytes, azurophils, heterophils, and basophils. Eosinophils are found in chelonians and lizards; however, their presence in snakes is not sufficiently studied. Snake lymphocytes are mononuclear cells and smaller than erythrocytes; the nucleus has a low standard of dense chromatin; the cytoplasm is basophilic; the number increases in circulation during inflammatory processes, wound healing, parasitemia, and viral diseases. The azurophils, the second most common leukocyte found in the blood of snakes, have a vacuolated cytoplasm and a central or eccentric nucleus; a number increase suggests the occurrence of infectious diseases. Heterophiles are large and eosinophilic and have eccentric nuclei and cytoplasmic granules that can be found intact or degranulated; a number increase is usually associated with an inflammatory response linked to inflammation, microbial and parasitic diseases, stress, and neoplasms. Basophils are small and spherical, with many granules in the cytoplasm. The function of snake basophils is probably the same as in mammals because such reptiles release immunoglobulins and histamine during degranulation [15].

Carvalho et al., 2017 [16] examined the leucocytes of *Boa constrictor*, *Bothrops jararaca*, and *Crotalus durissus* snakes. Cytochemistry and flow cytometry revealed small lymphocytes, large lymphocytes, azurophils, and heterophils. The authors did not detect any difference in the cell populations, but observed heterophils, lymphocytes, and azurophils with phagocytic activity [16]. Farag and El Ridi, 1986 [17] used spleen cells of the *Psammophis sibilans* adult snake to demonstrate that such lymphocytes can be stimulated by concanavalin A. Three years later, Saad, 1989 [18] used concanavalin A, phytohemagglutinin, and *Escherichia coli* lipopolysaccharide as a mitogen to show that mitogenic responsiveness of such snake lymphocytes varies according to the animal’s sex.

There are reports of hemolysis tests indicating that the complement system of the *Naja kaouthia* snake’s innate immunity (actual species name of *Naja naja kaouthia*, Reptile Database [19]) is similar to that of mammals [20]. Such a complement cascade seems to act in two ways: (1) direct adherence to microbial cell membranes without any involvement with the adaptive immune system; or (2) direct pathogen lysis via the formation of a membrane attack complex that perforates pathogen cell membranes [21].

On the other hand, AMPs are also part of innate immunity. Among them, the best known are cathelicidins and defensins, which belong to the large group of cationic peptides with amphipathic properties. Such a group corresponds to the main part of the host defense in many vertebrates [22], and includes peptide chains of low molecular weights (MW) or short AMPs with antibiotic activity. All these types of AMPs will be further discussed below.

Most published studies on innate immunity in snakes used samples of their plasma for tests on vertebrate erythrocytes aiming to verify the complement activity [23] and lysis of the Gram-negative (G−) bacteria *Escherichia coli*, the Gram-positive (G+) *Staphylococcus aureus*, and the fungus *Candida albicans* [24]. This approach has been widely explored in ecological studies involving snakes, with the results indicating the immunity of reptiles is closely dependent on several intrinsic factors related to the snake or the environment [25]. This type of result and the mitogenic responsiveness of lymphocytes has helped to evaluate the immune capacity of snakes (Table 1). Indeed, studying several mesic snake communities, Brusch et al., 2020 [26] found a correlation between dehydration and the presence of hemoparasites with cellular and humoral immunity.

## 3. Antimicrobials Related to Snake Venoms

In 1991, Stiles [47] published a systematic work showing that venoms of 30 Elapidae and Viperidae snakes were active against G− (*Aeromonas hydrophila*, *Pseudomonas aeruginosa*, *Escherichia coli*) and G+ (*Staphylococcus aureus*, *Bacillus subtilis*) bacteria. In addition, the authors observed that L-amino acid oxidase (LAAO) was the main toxin of *Pseudechis australis* venom with antibacterial activity [47]. Nonetheless, the first purified toxin tested against bacteria was an LAAO found in *Crotalus adamanteus* venom by Skarnes, 1970 [48]. Since then, antimicrobial activities have been detected on crude snake venoms, fractions of it, or in purified components [49].

### 3.1. Toxins—Proteins and Enzymes

In general, the macromolecules produced by living organisms as part of their innate immunity that are capable of inhibiting the growth of, or even killing, microorganisms pathogenic to them, acting as broad-spectrum anti-infectives, belong to the following families of proteins: lectins, metalloproteinases, LAAO, serine proteinases, and phospholipase type A_2_ (PLA_2_) [50]. See below a brief discussion of the members of each family.

#### 3.1.1. Lectins

Lectins from snake venoms are divided in two classes: C-type, or calcium-dependent, lectins that bind carbohydrate groups (true CTLs) and C-type lectin-like proteins (CLPs) not able to bind sugars [51]. Convulxin (CVX) is a heterodimeric toxin CLP isolated from the venom of South American rattlesnake *Crotalus durissus terrificus*, whose subunits α (CVXα, 13.9 kDa) and β (CVXβ, 12.6 kDa) are joined by inter- and intrachain disulfide bonds arranged in a tetrameric α4β4 conformation; CVXs activate platelets [52].

Historically, crotacetin (CTC), which is a CVX-like purified from the venom of *C. d. terrificus* [53], was the first of its family described as having antibacterial activity. At 150 µg/mL, both CVX and CTC can inhibit the cellular growth of the G− bacteria *Xanthomonas axonopodis* pv. passiflorae and *Clavibacter michiganensis michiganensis* by 87.8% and 96.4%, respectively. Interestingly, the monomeric subunits of these antimicrobial proteins do not display any antibacterial activity [53]. 

The homodimer of 33.6 kDa BpLec was isolated from *Bothrops pauloensis* and reported as an efficient inhibitor of *S. aureus* (G+) growth at an MIC of 31.25 µg/mL, although it was not able to affect *E. coli* (G−) growth even after 22 h of incubation [54].

In 2011, Nunes et al. [55] described BlL, a CLP isolated from *B. leucurus* snake venom that has molecular mass of 30 kDa, is composed of two subunits of 15 kDa, and showed activity against the human pathogenic G+ bacteria *S. aureus*, *Enterococcus faecalis*, and *B. subtilis* (with MICs of 31.25, 62.25, and 125 μg/mL, respectively), but not against the G− bacteria *E. coli* and *Klebsiella pneumoniae*. These data suggested that although lectins can interact with the peptidoglycan present in the cell wall of G+ bacteria, they cannot cross the outer membrane of G− bacteria to reach the periplasmic space. Since BlL showed no antimicrobial activity in the presence of 200 mM galactose, this result indicated that its antibacterial effect involves the carbohydrate-binding property of lectin.

Six years later, Sulca et al. purified another CLP (14/18 kDa) from *Bothriopsis oligolepis*, active against *S. aureus* (G+) ATCC 25923 with an MIC of 100 µg/mL [49], so the authors digested it by incubation with highly purified bovine pancreatic trypsin to search for new AMPs among the resulting peptide fragments. 

It was also reported that a CLP from *B. jararacussu* venom did not affect bacterial growth, but was able to inhibit the formation of biofilms of *E. coli* (G−) and *Streptococcus agalactiae* (G+) and disrupt pre-formed staphylococcal biofilms of the G+ bacteria: *S. chromogenes*, *S. hyicus*, and *S. aureus* [56]. 

#### 3.1.2. Metalloproteinases

Zn^2+^-dependent snake-venom metalloproteinases (SVMPs) are specific hemorrhagic toxins derived from the disintegrin A and metalloproteinase (ADAM) cellular family. These enzymes are secreted, single-pass transmembrane proteins [57,58].

SVMPs of the PIII group are the closest homologs of cellular ADAMs because they are large multidomain toxins (60–100 kDa) containing an N-terminal metalloproteinase, a C-terminal disintegrin-like, and cysteine-rich domains. The members of the PII group (30–60 kDa) contain a disintegrin domain at the carboxyl terminus of the metalloproteinase domain. However, PI-metalloproteinases (20–30 kDa) are single-domain proteins. As members of a broad family of proteins formed by 40–100 amino acid (AA) residues, the disintegrins are cysteine-rich polypeptides isolated from the venoms of vipers and rattlesnakes. These proteins can be released in viper venoms by the proteolytic processing of PII SVMP precursors or biosynthesized from short-coding mRNAs [58].

Samy et al. [59] described a viper metalloproteinase (AHM) of *Gloydius halys* (actual name of the species *Agkistrodon halys* Pallas [19]) venom with antimicrobial activity. Once purified, this AMP was characterized as a single-chain polypeptide with a MW of 23.1 kDa, highly similar to other SVMPs present in Viperidae venoms, with antibacterial activity against *S. aureus* (G+, MIC >7.5 µM), *Burkholderia pseudomallei* (also known as *Pseudomonas pseudomallei*, G−, 30 µM), *Proteus vulgaris* (G−, 15 µM), *E. coli* (G−, 60 µM), *P. aeruginosa* (G−, 60 µM), and *Enterobacter aerogenes* (G−, 60 µM). Data obtained in scanning electron microscopy studies indicated that the protein interacts with the peripheral cell wall, causing an explosion-like disruption of the plasma membrane in G+ bacteria [59]. 

No activity against G+ bacteria has been reported for SVMPs up to 2017, when the research group of Institute of Chemistry-USP isolated and purified a PIII-SVMP (73/60 kDa) from *B. oligolepis*, with an MIC of 20 µg protein/mL against *S. aureus* ATCC 25923 [49]. Sulca-López et al. also found out that one of its tryptic peptide fragments could be modified to produce very effective AMPs active against a few species of *Candida* [49].

It should be mentioned that proteolysis of a *Cerastes cerastes* SVMP generated a disintegrin (1 mg) that can significantly inhibit (84.7%) the growth of the parasite *Leishmania infantum*, a flagellate protozoan and an etiologic agent of visceral leishmaniasis [60].

#### 3.1.3. Serine Proteinases

Snake-venom serine proteinases (SVSPs) are among the best characterized. These enzymes have molecular weights varying from 26 kDa to 67 kDa and various levels of glycosylation [61]. Because SVSPs act on various components of the vertebrate coagulation cascade on the fibrinolytic and kallikrein-kinin systems, they were further denominated as snake venom thrombin-like enzymes (SVTLEs). As to structure, the 30 members of this group share the active site sequence motif. A good example is the serine proteinase found in many snake venoms that resembles, at least in part, thrombin [62].

So far, SVSPs have not been associated with antimicrobial activity. Nevertheless, in 2017, Sulca et al. purified one (27 kDa) from *B. oligolepis* venom with an MIC of 80 µg/mL against *S. aureus* ATCC 25923 (G+) [49].

#### 3.1.4. L-Amino Acid Oxidases (LAAO)

These enzymes are classical flavonoid-containing proteins that catalyze the oxidative deamination of L-amino acids to convert them into keto acids, ammonia, and hydrogen peroxide (H_2_O_2_) [63]. The content of LAAO in snake venoms varies from 1% to 30% of all proteins [63,64,65].

As presented in Table 2, svLAAO exhibit antimicrobial activity, as they can inhibit the growth of both G− and G+ bacteria at different concentrations or amounts. It is highly accepted that this biological action is a consequence of H_2_O_2_ production during the aerobic oxidation of appropriate substrates, an explanation reinforced by the observation that catalase inhibits the antimicrobial activity of LAAO [66].

#### 3.1.5. Phospholipases A_2_ (PLA_2_)

Snake-venom PLA_2_s can be found in Elapidae and Viperidae snakes and are grouped according to the amino acid sequence (primary structure) and the pattern of disulfide bonds (tertiary structure), as Group I and Group II, respectively [86,87]. They can present as neurotoxic, myotoxic, or both [88]. Group II of PLA_2_s presents mainly in Viperidae venoms, shows myotoxic activity, and can be divided into Asp49- or Lys49-PLA_2_, the last being enzymatically inactive [86]. Most PLA_2_s from snake venoms have a basic character [87] in Viperidae snakes, and correspond to 40–50% of the dry weight of *Crotalus durissus terrificus* venom; it is the main responsible of crotalic venom toxicity [87]. Despite this low toxicity [88], an acidic PLA_2_ purified from the venom of *Porthidium nasutum* showed an antibacterial activity against *S. aureus* but not against *E. coli* [88], exposing the importance of the net charge to the antibacterial spectrum. It has been proposed that these phospholipases can inhibit bacterial growth by damaging the cell membrane’s lipid bilayer [89]. Unfortunately, the Asp49-PLA_2_ myotoxin cited above also causes myonecrosis and kidney failure in mammals, so this enzyme has not been considered a potential antibacterial agent [90]. A table listing other PLA_2_ from snake venoms with antimicrobial activity is shown below (Table 3).

Crotoxin, a *C. d. terrificus* PLA_2_, shows in vitro activity against yellow fever virus (EC_50_ of 0.04 ng/µL), dengue virus 2 (EC_50_ of 0.05 ng/µL) [91,92]. *B. asper* PLA_2_ was shown to be active against dengue virus at 1.7 ng/mL (IC_90_) as well as Rocio, Mayaro, and Oroupouche viruses (0.0021–0.0078 ng/mL, EC_50_) [93], prevented the release of HIV-1 strains (ID_50_ of 1 nM) [94], and inhibited the replication of the hepatitis virus C at 6.08 µg/mL (IC_50_) [95]. In addition to the antimicrobial action, the PLA_2_ of *B. jararacussu* displayed antitumoral activity [96].

**Table 3:** Antimicrobial activity of snake-venom phospholipases A_2_ (PLA_2_).

Snake	Microorganisms	Properties	Reference
*Atropoides nummifer*	*Salmonella typhimurium* (G−)	50 µg/mL (>80% inhibition), Lys49-PLA_2_	[97]
*Bothriechis schlegelii*	*S. typhimurium* (G−)	50 µg/mL (>50% inhibition), Lys49-PLA_2_	[97]
*Bothrops asper*	*S. typhimurium* (G−)	100 µg/mL (>50% inhibition), mt-I, II, III, and IV, Lys49-PLA_2_	[97]
*B. brazili*	*Escherichia coli (G−)*	120 µg/mL of Asp49-PLA_2_ and Lys49-PLA_2_ (80% of bacterial inhibition)	[89]
*B. jararacussu*	*E. coli* (G−)	5 µg/mL (>50% inhibition), Lys49-PLA_2_	[98]
*B. jararacussu*	*Xanthomonas axonopodis pv passiflorae* (G−)	125 µg/mL (<86% inhibition) BthTx-I, BthTx-II (Lys49-PLA_2_)	[99]
*B. marajoensis*	*Staphylococcus aureus* (G+)	50 µg/mL (MIC), Lys49-PLA_2_	[72]
*B. neuwiedi* (actual of *B. neuwiedi urutu*) *	*Pseudomonas aeruginosa* (G−)	100 µg/mL (60% inhibition), Lys49-PLA_2_	[100]
*Bungarus fasciatus*	*E. coli* (G−), *S. aureus (G+)*	0.4 and 0.1 µM (MIC), Group I PLA_2_	[101]
*Bungarus multicinctus*	*E. coli* (G−)	50 µM (80% inhibition), Group I PLA_2_	[102]
*Cerrophidion godmani*	*S. typhimurium* (G−)	100 µg/mL (>50% inhibition), mt-I and mt-II, Asp49-PLA_2_	[97]
*Crotalus adamanteus*	G+: *S. aureus*, G−: *Burkholderia pseudomallei*, *Enterobacter aerogenes*	7.8–15.6 µg/mL (MIC) wound healing in vivo by topical application	[103]
*C. durissus collilineatus*	*X. axonopodis pv. Passiflorae* (G−), *Clavibacter m. michiganensis* (G+)	250 µg/mL (>90% inhibition), Lys49-PLA_2_ with high enzymatic activity	[104]
*C. d. ruruima*	*X. axonopodis pv. passiflorae* (G−)	75 µg inhibit about 96% of the bacterial growth, Lys49-PLA_2_	[105]
*C. d. terrificus*	*B. pseudomallei* (G−)	0.5 mg/mL (radial diffusion), Asp49-PLA_2_	[106]
*C. d. terrificus*	G+: *S. aureus*; G−: *E. aerogenes*, *P. aeruginosa*, *E. coli*	100 µg/mL (radial diffusion), Asp49-PLA_2_	[107]
*C. oreganus abyssus*	G+: MRSA; G−: *P. aeruginosa*, *E. coli*	At 125 µg/mL inhibits 25–60% the bacterial growth, Lys49-PLA_2_	[108]
*Daboia russellii* (actual of *D. russellii pulchella)* *	G+: *S. aureus*, *Bacillus subtilis*; G−: *E. coli*, *S. typhimurium*, *Vibrio cholerae*, *Klebsiella pneumoniae*, *S. paratyphi*	12–15 µg/mL (MIC), VRV_PL_V, basic PLA_2_	[109]
*Daboia russellii* (actual of *D. russellii pulchella*) *	G+: *S. aureus*, *B. subtilis*; G−: *E. coli*, *S. typhimurium*, *V. cholerae*, *K. pneumoniae*, *S. paratyphi*	11–19 µg/mL (MIC), VRV-PL-VIIIa, basic PLA_2_	[110]
*D. russelli* (actual of *D. russelli russelli*) *	G−: *E. coli*, *E. aerogenes*, *Proteus vulgaris*, *P. mirabilis*, *P. aeruginosa*, *B. pseudomallei*; G+: *S. aureus*	6.25–100 µg/mL (MBC), VipTx-I and VipTx-II, Asp49-PLA_2_	[111]
*D. siamensis* (actual of *D. russellii siamensis*) *	*B. pseudomallei* (G−)	0.5 mg/mL (radial diffusion), basic PLA_2_	[106]
*D. siamensis* (actual of *D. russellii siamensis*) *	*S. aureus* (G+)	100 µg/mL (radial diffusion), basic PLA_2_	[107]
*Echis carinatus*	G−: *E. coli*, *E. aerogenes*, *P. vulgaris*, *P. mirabilis*, *P. aeruginosa*, *B. pseudomallei*; G+: *S. aureus*	15–60 µg/mL (MIC), Asp49-PLA_2_	[112]
*Lachesis muta muta*	G+: MRSA; G−: *P. aeruginosa*, *K. pneumoniae*	12.5 µg/mL of Lys49-PLA2 named LmutTX, inhibits about 60% of G+ and ~30–50% of G− bacteria	[113]
*Montivipera bornmuelleri*	G−: *E. coli*, *P. aeruginosa*; G+: *S. aureus*	100 µL of no informed concentration (radial diffusion), the type of PLA_2_ was not informed	[114]
*Naja naja*	G+: *S. aureus*, *B. subtilis*; G−: *E. coli*, *S. typhi*, *V. cholerae*, *K. pneumoniae*, *S. paratyphi*, *P. aeruginosa*; *C. albicans*, *Trichophyton tonsurans*	19–23 µg/mL (MIC), NN-XIa-PLA_2_, acidic PLA_2_	[115]
*Naja naja*	G+: *S. aureus*, *B. subtilis*; G−: *E. coli*, *S. typhi*, *V. cholerae*, *K. pneumoniae*, *S. paratyphi*	17–120 ug/mL (MIC), NN-XIb-PLA_2_, acidic PLA_2_	[116]
*Porthidium nasutum*	*S. aureus* (G+)	32 µg/mL (MIC), acidic PLA_2_	[88]
*Pseudechis australis*	*B. pseudomallei* (G−)	0.5 mg/mL (radial diffusion), Group I PLA_2_	[106]
*P. australis*	*E. aerogenes* (G−)	100 µg/mL (radial diffusion), Group I PLA_2_	[107]

Microorganisms, microorganisms sensitive to antimicrobial activity; MIC, minimum inhibitory concentration; MBC, minimal bactericidal concentration; MRSA, methicillin-resistant *S*, *aureus*; G−, Gram-negative bacteria; G+, Gram-positive bacteria; * the actual species name was consulted in the Reptile Database [19].

#### 3.1.6. Cysteine-Rich Secretory Protein (CRISP)

The protein crovirin with 24.9 kDa was purified from *C. viridis viridis* venom. It was active on different forms of *Trypanosoma cruzi*, *T. brucei rhodesiense*, and *L. amazonensis* with IC_50_ ranging from 1.10 µg/mL to 2.38 µg/mL [117].

Finally, Table 4 presents other snake-venom protein toxins active on fungi and parasites not presented in the previous tables.

### 3.2. Oligopeptides with ≥60 Amino Acid Residues

#### 3.2.1. Waprins

These oligopeptides or small proteins show structural similarity to whey acidic proteins (WAPs). Omwaprin, whose structure contains 50 AA residues and four disulfide bridges, was purified from *Oxiuranus microlepidus* venom [129]. Recombinant omwaprin has been produced and tested in a radial diffusion assay; the results revealed activity against the G+ bacteria *B. megaterium* (560.2 μg/mL) and *S. warneri* (1.7 mg/mL), but not against G+ strains of *B. thuringiensis*, *S. aureus*, and *Streptomyces clavuligerus*, or G− strains of *E. coli* (BL21) and *Agrobacterium tumefaciens* (even at the dose of 5.6 mg/mL). This AMP is also reported as relatively salt tolerant (as it was active on bacteria even at 250 mM NaCl), not hemolytic up to 1 mM, and not toxic to Swiss albino male mice at concentrations up to 10 mg/kg. It specifically targets bacterial membranes.

As nawaprin is a very similar structure isolated from the venom of *Naja nigricolis* [130], it also belongs to the waprins family and was expected to display antibacterial activity but, so far, no results have confirmed such ability.

#### 3.2.2. Cardiotoxins

Three-finger toxins are members of a family of highly basic small proteins (MW of approximately 6.5 kDa) commonly found in elapid venoms. Among them are the cardiotoxin produced by *Naja atra* (actual species name of *N. naja atra* [19]) [131] and *Naja nigricolis* gamma toxin [132] that, beyond the cardiotoxicity, are active against *E. coli* (G−) and *S. aureus* (G+). The fusogenic effect on phosphatidylethanolamine (PE)/phosphatidylglycerol (PG) and PG/cardiolipin vesicles of both toxins has been used to explain their antibacterial activity [133].

#### 3.2.3. Peptide VGF-1

Isolated from *Naja atra* venom, this toxin formed by 60 AA residues inhibits the growth of drug-resistant clinical strains of *Mycobacterium tuberculosis* (G+) at the concentration of 8.5 mg/L [12].

### 3.3. Peptides Containing 2-58 Amino Acid Residues

Most naturally occurring AMPs contain 2-50 AA residues; they are cationic compounds owing to the presence of one or some arginine and lysine residues and, consequently, they have net charges varying from +2 to +6 at a neutral pH. The majority are composed of amphiphilic sequences, meaning that in solution, these AMPs can acquire secondary structures, especially amphipathic α-helices typically characterized by a hydrophobic face exhibiting non-polar AA residues and a hydrophilic face displaying polar or positively charged amino acids [2,8,134].

As already cited, such AMPs inhibit bacterial and fungal growth, and many also kill these microorganisms at low minimum concentrations by different molecular mechanisms of action. Most of these antimicrobials are cell-membrane active, meaning that they act through the disruption or permeabilization of such cellular targets. It has been proposed that this phenomenon occurs by three non-exclusive types of events: detergent action or micellization (carpet model), barrel stave pore formation, and toroidal pore formation. The other possible events are disordered toroidal pore formation, membrane thinning/thickening, charged lipid clustering, formation of non-bilayer intermediate, oxidized lipid targeting, involvement of an anion carrier, non-lytic membrane depolarization, and electroporation. It follows comments on naturally occurring AMPs of low MW [135].

#### 3.3.1. Pep5Bj

Pep5Bj is present in *B. jararaca* venom, with 1370 Da, is active against the phytopathogenic fungi *Fusarium oxysporum*, *Colletotrichum lindemuthianum*, and against the yeasts *Candida albicans* and *Saccharomyces cerevisiae* [136].

#### 3.3.2. β-Defensins

The first β-defensin found in snakes was crotamine, a small basic myotoxin from the venom of the rattlesnake *C. d. terrificus*. It contains 42 AA residues and presents a net charge of +7 at a neutral pH and a motif of six cysteines, characteristic of this AMP family [137]. Crystallography followed by X-ray diffraction indicated that such an AMP structure is organized in a β-sheet-rich fold with a three-dimensional (3D) structure similar to β-defensins, as confirmed by Coronado et al. [138].

Crotamine is a myotoxin that acts on negatively charged plasma membranes, causing bursts to giant unilamellar vesicles (GUVs) [139] and in *E. coli* (G−), *Citrobacter freundii* (G−), *B. subtilis* (G+), and *Micrococcus luteus* (G+) cells [140,141,142]. It also inhibits the growth of *Candida* spp, *Trichosporon* spp, and *Cryptococcus neoformans* [123], as well as displays antiplasmodial activity, here exemplified by the IC_50_ of 1.87 µM found for *Plasmodium falciparum* [124].

Genomics-based approaches have been used to discover genes of innate immunity related to this group of AMPs [143,144]. Although mature β-defensins have a high variation in the AA sequence, it is known that the untranslated regions and signal peptides are highly conserved. Depending on the snake family, the propeptides are codified in two exons (Boidae, Elapidae, and Colubridae snakes) [145] or three exons (Viperidae snakes) [146]. So, due to the small size of β-defensin genes, the PCR approach was shown to be the most suitable for phylogenetic analysis of β-defensin-like genes in pit vipers [146] and colubrid snakes [145]. Crotamine-like genes identified in Brazilian pit vipers were used to deduce the amino acid sequences codified in the exons, and design and synthesize linear peptides with approximately 4 kDa. They were capable of inhibiting the bacterial growth of *E. coli* (G−), *C. freundii* (G−), *M. luteus* (G+), and *S. aureus* (G+) with MICs ranging from 1.6 µM to 28.4 µM [142].

**Figure 1:**
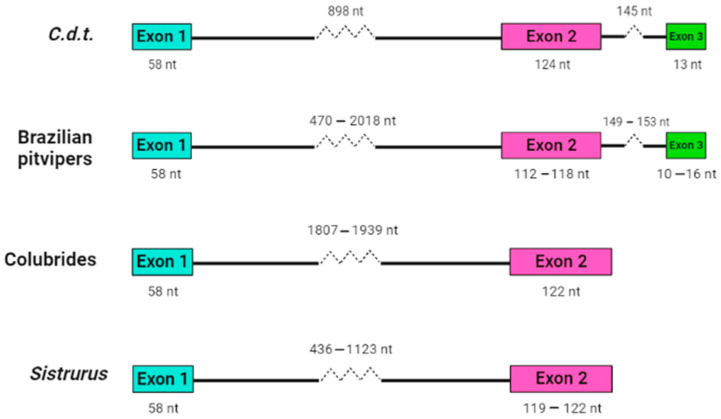
Structural organization of snake β-defensin genes. Crotamine sequence (*C.d.t.*, GenBank AF223947 [149]), crotamine-like sequences of Brazilian pit vipers [146], β-defensin-like sequences of Colubrides (*Phalotris mertensi*, *Thamnodynastes hypoconia*, and *T. strigatus* [145], and crotamine-like sequences of *S. miliarius* (GenBank MT024631-024638). Only exons and introns are represented.

Our research group working at Instituto Butantan analyzed crotamine-like sequences of *Sistrurus catenatus* and *S. miliarius*, rattlesnakes from the USA [147], using an approach very similar to that developed by Corrêa and Oguiura [146]. The DNA of North American rattlesnakes was used as a template in PCR, sequences were concatenated using Geneious software [148] as described in the Supplementary Materials. Although it was impossible to amplify crotamine-like sequences of *S. catenatus*, the authors analyzed eight sequences from *S. miliarius* derived from two specimens of Florida (accession number MT021631-024638 on GenBank) and found that the propeptides are encoded in two exons and can be grouped into two sets, one with a short intron with approximately 400 bp and the other with a long intron with about 1100 bp. The introns are phase 1 (inserted after the first nucleotide of codon), as are those of other snake β-defensins. The sequences with a short intron (MT024631-02633) codified only one β-defensin sequence. Such gene organization (Figure 1) is similar to the β-defensin genes of the Colubridae, Boidae, and Elapidae snake families [145], but not of the pit vipers [146].

**Figure 2:**
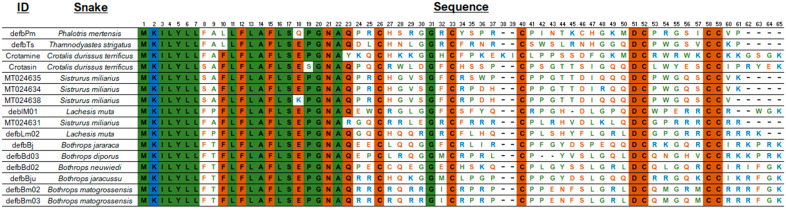
Amino acid sequences of snake β-defensins. Alignment used MUSCLE [150]), and the figure edition employed BioEdit [151] and the BioRender was used to create the art. Non-polar amino acid residues are in green, positively charged amino acid residues in blue, and the polar amino acid residues, including cysteines, glycines, and prolines, in brown.

The alignment of the AA sequences (Figure 2) shows a conserved signal peptide, the motif GNA, and the cysteine residues that determine the 3D β-defensin structure as well as the glycine residue at position 31. Interestingly, mature *S. miliarius* β-defensins have glutamine as first amino acid, as have the other snake β-defensins described, except for MT024631, which begins with an arginine.

**Figure 3:**
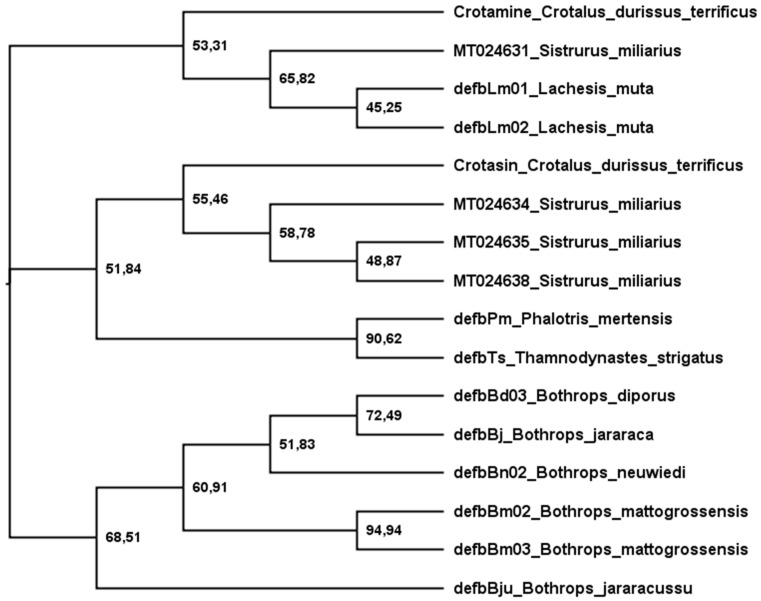
Phylogenetic tree of snake β-defensins. The tree was estimated using translated sequences and maximum likelihood [153]. The Edge LR-ELW support is shown in each node [154]. Details are described in the Supplementary Materials.

Figure 3 shows a phylogenetic tree of snake-venom β-defensins built after analyses using maximum likelihood. The sequences were grouped into three main branches: (1) crotamine-like, (2) crotasin-like, and (3) *Bothrops*. (1) The crotamine-like group constitutes sequences of crotamine and *Lachesis* β-defensins that are active against *E. coli* (G−), *M. luteus* (G+), *C. freundii* (G−), and *S. aureus* (G+). Crotasin is a paralogous gene of crotamine found in South American rattlesnakes [152] with no antibacterial activity [142], so (2) crotasin-like group encompasses crotasin, *Sistrurus* sequences closely related to crotasin, and colubrid sequences with antibacterial activity against only *M. luteus* (G+) [142]. (3) The *Bothrops* group shows three subgroups: the DefbBju with no antibacterial activity, the *B. mattogrossensis* sequences with the highest antibacterial activity and active against *E. coli* (G−), *M. luteus* (G+), *C. freundii* (G−), and *S. aureus* (G+). In the remaining subgroup, while DefbBd03_B. diporus and DefbBj_B. jararaca show activity only against *M. luteus* (G+), DefbBn_B. neuwiedi has no antibacterial activity [142]. Of the four translated sequences of *Sistrurus*, only one (MT024631) was grouped with crotamine and the others were grouped with crotasin. Interestingly, in the crotasin group, while the *Sistrurus* sequences (MT024634, MT024635, MT024638) have net charges at pH 7 of +1; in the crotamine group, MT024631 has +11. The MT024631 position in the phylogenetic tree and its high basicity makes this sequence a strong candidate for exhibiting high antimicrobial activity.

#### 3.3.3. Cathelicidins (CATH)

These peptides are multifunctional biomolecules resulting from the propeptide proteolytic cleavage [155]. The first ones discovered were isolated from venoms produced by Asian elapid species [156], including *Bungarus fasciatus* [121] and *Ophiophagus hannah* [156]. These bioactive peptides are members of a group of AMPs that present variations in their amino acid sequences, chemical structures, and sizes. On the other hand, they all have in common two functional domains: one of them has high homology to the cathelin domain from which the name cathelicidins originated, a well-known inhibitor of cathepsin L; the other domain is the antimicrobial one, located at the C-terminus of the structure, also presents wide functional diversity [22,157]. The antimicrobial domains of some cathelicidins have α-helical conformations, others have β-hairpin structures and might contain high content of proline and arginine. Even though the mature peptides contain 12 to 80 or more AA residues, some discussed here contain 30–34 [158].

All CATH are encoded by genes that are made up of four exons [158]. The first exon consists of the sequence encoding the signal peptide (pre-peptide) of 29–30 AA residues, while exons 2 and 3 encode the cathelin domain (pro-peptide) of 99–114 AA residues. Exon 4 encodes the mature peptide, with the antimicrobial domain [158]. Cathelicidin genes have not been described in snakes, but Dalla Valle et al. [159] demonstrated that the genes of the lizard *Anolis carolinensis* have structural organization similar to that of mammals, which is up to four exons with three introns of different sizes. Mature cathelicidins generally exhibit antimicrobial activity against a wide range of Gram+ and Gram- bacterial species [160]. These antimicrobial peptides and proteins were found in transcripts of venom glands and others in genomes.

As experiments using NA-CATH and liposomes have shown, the main event of the general mechanism of action proposed for cathelicidins is the disruption of the bacterial cell membrane [161,162]. However, elapid venom cathelicidins can also inhibit *E. coli* ATP synthase [163]. Their low MICs for Gram+ and Gram- bacteria, resistance to salt and serum, and in vivo activity make these macromolecules promising candidates for new antimicrobial drugs. Further information on 13 different CATH antimicrobials is summarized in Table 5 and in the review published by Barros et al. [164]. 

Cathelicidins also display anti-inflammatory activity that helps the recovery of organisms with pneumonia [172], other inflammatory diseases [184,185], and pathogen-induced intestinal injury [186]. In vivo, Cath-BF was found to help treat burn and wound infections in rats [11], and protect mice against sepsis caused by *E. coli* (G−), *P. aeruginosa* (G−), and *S. aureus* (G+) [187]. In addition, Cath-BF inhibited intestinal inflammation and enhanced the phagocytosis of immune cells in weanling piglets [186].

**Figure 4:**
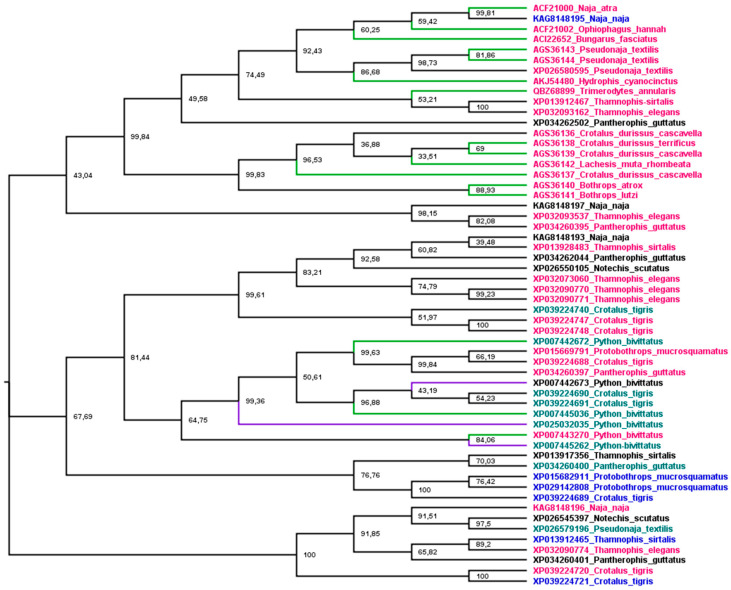
Phylogenetic tree of snake cathelicidins. The tree was estimated based on maximum likelihood [153], and the Edge Support LR-ELW is shown in each node [154]. Details are described in the Supplementary Materials. Branches in green indicated sequences with antimicrobial activity, purple branches indicate no activity, and black ones were not tested. Sequence names in purple indicate net charge < 5, in green 5 < 10, red > 10, and black, not determined, except XP007442673, which shows −4 as net charge at pH 7.

Phylogenetic analysis was used to understand the relationship between snake cathelicidins (Figure 4). The cathelicidin sequence tree did not group in species or family snakes. This disconnection between the species tree and the sequence tree is due to the duplications and extinctions that the genes of multigenic families undergo [188]. The tree is grouped into three main branches. The most basic group (1) presents an exception, KAG8148195, with a net charge of +4, and all cathelicidins tested in this group showed antibacterial activity. The second group (2) encompasses an extensive range of net charges and *Python bivittatus* cathelicidins with and without antibacterial activity. The last group (3), with a wide range of net charges, did not have any member tested. Group 1 shows three subgroups, two with Elapidae and Colubridae snake sequences and one with Viperidae. The association of Elapidae and Colubridae sequences was observed in snake β-defensins [145]. Group 2 is also organized into three subgroups, but there is no Viperidae branch (*Crotalus* CATHs are present in all subgroups). Moreover, all *P. bivittatus* sequences are associated in one subgroup independently of antibacterial activity. The last group assembled was not tested for cathelicidins of any family snake.

#### 3.3.4. Peptides Derived from Larger AMPs from Snake Venoms (Proteins and Oligopeptides)

Short and medium-sized peptides with pharmacological functions have been widely studied, owing to their potential to become therapeutic drugs or serve as lead compounds for developing new ones. Indeed, such short biopolymers can be much more specific to cellular targets than other non-peptide drugs. On the other hand, in vivo they are prone to enzymatic degradation, can be sensitive to high salt concentrations, be cytotoxic, or interfere with the host immunity. These disadvantages have been extensively studied in order to overcome these problems: mutations and/or modifications of their reactive chemical groups have been tested [189].

Table 6 lists several short AMPs found in snake venoms that correspond to peptide fragments of snake toxins with the ability to inhibit the growth and even kill a variety of pathogenic microorganisms. As the table shows, these peptides represent specific portions of proteins, enzymes, or oligopeptides with the antimicrobial activity described above, such as cathelicidins, myotoxins, PLA_2_, and defensins, unmodified or modified. A comparative analysis of their amino acid sequences reveals that practically all are cationic at a neutral pH and, as do most of the short cationic AMPs already described, have amphiphilic structures. 

Among the many examples given is Ctn(15–34), a fragment of 20 AA residues from the 34-mer Crotalicidin, able to kill Gram- and Gram+ bacteria [201,203]. Clinical isolates of fungi were tested associated with fluconazol and presented additive activity [212], as well as damaged tumor cells [213]. Ctn(15–34) also has remarkable stability in human serum, is regarded as a promising anti-infective lead compound, and its mode of action seems to comprise the three stages needed for membrane-active AMPs: (1) initial peptide recruitment; (2) peptide accumulation on the phospholipidic bilayer of the plasma membrane; and (3) cell death caused by disruption of the plasma membrane.

The M.T. Machini research group (Institute of Chemistry-USP) has been developing new short AMPs active against *Candida* species derived from fragments of a metalloprotease and a PLA_2_ found in the venom of the Peruvian snake *B. oligolepis*, still very little studied [49].

## 4. Discussion

This review shows that the innate immunity of snakes is similar to that of mammalian vertebrates in terms of cell-mediated and humoral responses. The blood of these animals contains erythrocytes, thrombocytes, and leukocytes [15], and the lymphocytes have phagocytic activity [16]. Snake immunity can be influenced by hormones, daily and seasonal rhythms, temperature, and dehydration, as shown in Table 1. These factors have been widely studied with an ecological focus using plasma samples. Since their influence on innate immunity does not interfere with snakes’ adaptive capacity, these reptiles have spread to different ecosystems and microhabitats.

The ability of snakes to live in different environments, to resist different pathogenic microbes, and to eat different prey makes their venom a rich source of biomolecules that can be explored as a biological tool for science or potential anti-inflammatory, analgesic, antitumor, or antimicrobial agents. The venom has a potent antimicrobial activity, so snakes can keep their prey uncontaminated when digestion takes days.

One of the major problems facing public health is the growing resistance of microbes to antibiotics, so multiple scientific approaches have been employed to find new antimicrobials with high therapeutic indexes. Natural secretions, including snake venoms, have been considered excellent sources of bioactive compounds, with mechanisms of biological and physiological actions alternative to those of the conventional antibiotics. Thus, these proteins, oligopeptides, and short peptides can be seen as potential bactericides and fungicides, or valuable leading molecules [214]. In addition, larger AMPs can be proteolyzed to generate short antimicrobial fragments. The information given here fully agrees with a previous report that also discusses this important matter [215].

In the last century, snake-venom toxins were extensively studied for their antimicrobial activity and other properties, most likely because they are an abundant natural source [216]. As emphasized here, the AMPs studied more recently are cathelicidins (Table 4, Table 5 and Table 6) and defensins. Indeed, with a few exceptions, these macromolecules can be expressed on demand in low or large amounts, and they fit the pattern described above. Transcriptome and genome databases can help to overcome any difficulty concerning obtaining biomolecules that have a low expression or that are not easily purified.

In this report, we also describe new sequences obtained from the genome of the rattlesnake *S. miliarius* using PCR. Eight were shown to codify four β-defensins, but only one peptide has antimicrobial potential as predicted by the phylogenetic analysis (Figure 3) and calculation of theoretical net charge. This peptide was encoded by MT024631, MT024632, and MT024633 sequences.

The association of phylogenetic analysis and biological activity can provide us with indications to choose the best organism for searching for the molecules that have the necessary biological activity or sequences and help select the best minimal structure to develop [217]. Such an approach was used for cathelicidins. Phylogenetic relationships were established, and the antimicrobial activities and net charges were associated with sequences. In this context, the phylogenetic tree of Figure 4 showing cathelicidin groups with antibacterial activity (1) with and without activities (2), and not tested (3) indicates that the unknown sequences with a larger chance of having antimicrobial activity could be those related to group 1. In order to confirm this hypothesis, more antimicrobial tests need to be done with the molecules of this branch.

Finally, this article reinforced that the peptides of snake venoms are valued biopolymers that could be used in vivo as antimicrobial drugs for activating the cellular and immune response of superior animals, and improving the immune response to infection. An interesting proposal is to employ mixtures of AMPs combined with conventional antibiotics, aiming to potentiate their actions on pathogenic microorganisms and circumvent drug resistance [197,205]. Snake-venom proteins, oligopeptides, and short peptides can also be used for wound healing, preventing infection, and increasing cell regeneration. 

Much remains to be done in this field of research after finding a new bioactive molecule, such as maintaining or increasing bioactivity under physiological conditions, decrease cytotoxicity, and increase chemical stability in vivo. The protection of peptides by carboxyamidation can increase the chemical stability and improve antimicrobial activity [9,205].

## 5. Conclusions

In conclusion, snakes and their secretions are important sources of antimicrobials. Molecular evolution and phylogeny approaches, in addition to traditional techniques such as proteomics, transcriptomics, peptide chemistry, and in silico studies, can increase the success of searching for new molecules with therapeutical potential or peptide-based lead compounds.

## Figures and Tables

**Table 1:** Plasma innate immunity and association to environmental and physiological conditions.

Snake	Microorganisms	Assay	Factors	Reference
*Agkistrodon piscivorus*	*E. coli* (G−)	Antimicrobial assay using plasma from cottonmouth pregnant relative to non-pregnant females.	Pregnancy	[23]
*A. piscivorus*	*E. coli* (G−)	Antimicrobial action of the complement system from plasma samples obtained in different seasons.	Temperature	[21]
*Antaresia childreni*	G−: *E. coli Salmonella enterica*.	Effect of the immune system against the growth of pathogenic G− bacteria in *A. childreni* eggs under normal environmental conditions of incubation and dehydration.	Dehydration	[27]
*Boa constrictor*	*E. coli* (G−)	Antibacterial activity of blood plasma from *Boa constrictor* fasting and fed with mice.	Feeding	[25]
*Crotalus atrox*	G−: *E. coli*, *S. enterica*	Bacterial killing assays of plasma to inhibit the growth of G− microorganism.	Dehydration	[28]
*C. durissus*	*E. coli* (G−)	Effects of temperature (25 °C to 35 °C) on antibacterial activity and variation of corticosterone levels in plasma.	Temperature	[29]
*C. viridis*	*G−: E. coli*, *Klebsiella oxytoca*, *S. typhimurium ^#^*, *Citrobacter freundii*, *Shigella flexneri*, *Enterobacter cloacae*; G+: *Streptococcus pyogenes*, *Staphylococcus aureus.*	The antimicrobial effect of *C. viridis*’s plasma against the growth of G+ and G− bacteria.	Temperature	[30]
*Liasis fuscus*	*E. coli* (G−)	Effect of dehydration conditions in adults, measuring osmolality of plasma and response of it against bacteria.	Dehydration	[31]
*Natrix piscator*	*Saccharomyces cerevisae*	Snake lymphocyte proliferation and phagocytosis against yeasts by snake macrophage.	Testosterone	[32]
*N. piscator*	*S. cerevisae*	Snake splenocyte proliferation and phagocytosis against yeasts by snake macrophage.	Daily and seasonal rhythms	[33]
*N. piscator*	*S. cerevisae*	Snake lymphocyte proliferation and phagocytosis against yeasts by snake macrophage.	Photoperiod	[34]
*Panterophis guttatus*	Not described	Hemagglutination on whole sheep blood.	Feeding	[35]
*Sistrurus miliarius*	Lipopolysaccharides (LPS) extracted from *E. coli* (G−)	Quantification of the metabolic cost in the immune response of pregnant and non-pregnant snakes using LPS.	Pregnancy	[36]
*S. miliarius*	*E. coli* (G−)	Relationship between environmental factors or the energetic/physiological state of snakes against infections.	Climate	[37]
*Thamnophis elegans*	*E. coli* (G−)	Comparison of the innate efficiency of the immune system in fast-living and slow-living snakes.	Size and age	[38]
*T. elegans*	*E. coli* (G−)	Hemolysis and hemagglutination on sheep red blood cells.	Ecotype and age	[39]
*T. elegans*	*E. coli* (G−), *S. aureus* (G+), *Candida albicans*	Realization of a new method for the microbicidal analysis of blood plasma by spectrophotometry.	Inter- and intraspecific variation	[24]
*T. elegans*	*E. coli* (G−)	The effect of fasting and stress on altered energy use and immune function.	Stress, food restriction	[40]
*T. elegans*	*E. coli* (G−)	Snake lymphocyte proliferation.	Pregnancy	[41]
*T. elegans*	*E. coli* (G−)	Snake lymphocyte proliferation.	Ecotype and parasitosis	[42]
*T. elegans*	*E. coli* (G−)	Interaction of immunological and endocrinological efficiency in inhibiting the growth of G− microorganisms.	Corticosterone, temperature, climate	[43]
*T. elegans*	*E. coli* (G−)	Effects of annual climate variation on immunity and bactericidal competence.	Climate	[44]
*T. sirtalis*	*E. coli* (G−)	Hemolysis on sheep red blood cell.	Physiology	[45]
*T. sirtalis*	*E. coli* (G−)	Effects of annual climate variation on immunity and bactericidal competence.	Corticosterone, temperature, climate	[43]
*T. sirtalis*	*E. coli* (G−)	Bacterial killing assays of plasma and hemolysis and hemagglutination on sheep red blood cells.	Climate	[44]
*Vipera ammodytes ammodytes*	Not described	Snake leukocyte proliferation, quantity of immune complexes and immunoglobulins.	Climate, shedding, hibernation, activity	[46]
*Vipera berus berus*	Not described	Snake leukocyte proliferation, quantity of immune complexes and immunoglobulins.	Climate, shedding, hibernation, activity	[46]

Microorganisms, microorganisms sensitive to snake plasma; assay, response of snake immunity cells to induction; factors, factors that influence innate immunity; G−, Gram-negative bacteria; G+, Gram-positive bacteria; ^#^ *Salmonella enterica* serovar typhi.

**Table 2:** Antimicrobial activity of snake-venom L-amino oxidase (LAAO).

Snake	Microorganisms	Properties	Reference
*Agkistrodon blomhoffii*	*Staphylococcus aureus* (G+)	>3.75 µg (standard disc-diffusion)	[67]
*A. halys* ^#^	*Escherichia coli* (G−), *Bacillus subtilis* (G+)	8.5 µg/mL (IC_50_)	[68]
*Bothriechis schlegelii*	*S. aureus* (G+), *Acinetobacter baumanni* (G−)	2–4 µg/mL (MIC); 4–8 µg/mL (MBC)	[69]
*Bothrops alternatus*	*E. coli* (G−), *S. aureus* (G+)	48 µg (kill 80–90% of bacteria)	[70]
*B. jararaca*	G+: *Eubacterium lentum*, *Propionibacterium acnes*, *S. aureus*, *S. epidermidis Peptostreptococcus anaerobius*; G−: *Porphyromonas gingivalis*, *Prevotella intermédia*, *Pseudomonas aeruginosa*, *S. typhimurium*	40 µg of venom (standard disc-diffusion)	[71]
*B. marajoensis*	*S. aureus (G+)*, *Pseudomonas aeruginosa* (G−), *Candida albicans*	>50 µg/mL (MIC) 2.55–2.86 µg/mL (IC_50_)	[72]
*B. mattogrosensis* (actually *Bothropoides mattogrosensis)* *	G+: *Bacillus subtilis*, *Enterococcus faecalis*, *S. aureus*, *Streptococcus pyogenes*; G−: *E. coli*, *Klebsiella pneumoniae*, *Proteus mirabilis*, *P. aeruginosa*, *S. typhimurium*	64–256 µg/mL (MIC)	[73]
*B. moojeni*	G+: *S. aureus*; G−: *P. aeruginosa*, *S. typhimurium*, *E. coli.*	50 µg/mL (inhibit <90% of cfu)	[74]
*B. paoloensis*	*E. coli* (G−), *S. aureus* (G+)	25 µg/mL (inhibit <90% of bacterial growth)	[75]
*B. pirajai*	G−: *E. coli*, *P. aeruginosa*	4 µg/mL (inhibit <90%)	[76]
*Calloselasma rhodostoma*	*E. coli* (G−), *S. aureus* (G+)	0.2–125 µg/mL (MIC)	[77]
*Crotalus adamanteus*	G+: *Micrococcus aureus*; G−: *S. typhimurium*, *P. vulgaris*, *P. aeruginosa*, *Serratia marcenses*, *E. coli*	1–50 µg (LD_50_, <50% inhibition of bacterial growth)	[48]
*C. durissus cascavella*	*Xanthomonas axonopodis pv passiflorae* (G−); *Streptococcus mutans* (G+)	12.3–35 µg/mL (50% of inhibition)	[78]
*C. d. cumamensis*	*S. aureus* (G+), *A. baumannii* (G−)	8–16 µg/mL (MIC)	[79]
*Daboia russellii*	G+: *S. aureus*; G−: *P. aeruginosa*, *E. coli*	4.5–36 µg/mL (MIC); 9–72 µg/mL (MBC)	[80]
*Naja oxiana* (actual of *Naja naja oxiana*) *	*B. subtilis* (G+); *E. coli* (G−)	0.036–0.094 µM (IC_50_)	[81]
*Ophiophagus hannah*	G−: *K. pneumoniae*, *P. aeruginosa*, *E. coli*; G+: *S. aureus*, *S. epidermidis*	0.78–50 µg/mL (MIC)	[82]
*O. hannah*	G+: *S. aureus*, *MRSA*, *S. epidermidis*, *B. subtilis*, *B. cereus*; G−: *S. enteritidis*, *P. aeruginosa*, *S. marcescens*, *K. pneumoniae*, *E. coli*, *Enterobacter cloacae*	7.5–110.0 µg/mL (MIC)	[83]
*Pseudechis australis*	G+: *B. subtilis*, *S. aureus*; G−: *Aeromonas hydrophila*, *A. sobria*	18 µg (standard disc-diffusion)	[47]
*Trimeresurus stejnegeri*	*HIV*	1.5 nM (EC_50_)	[84]
*Vipera lebetina*	*E. coli* (G−), *B. subtibilis* (G+)	20 µg/mL (MIC)	[85]

Microorganisms, microorganisms sensitive to antimicrobial activity; MIC, minimum inhibitory concentration; MBC, minimal bactericidal concentration; G−, Gram-negative bacteria; G+, Gram-positive bacteria; MRSA, methicillin-resistant *S. aureus*.

**Table 4:** Snake toxins activity against fungi and parasites.

Snake	Microorganisms	Properties and Toxin	Reference
*Bothrops asper*	*Plasmodium falciparum*	1.42–22.89 µg/mL (IC_50_), Asp49-PLA_2_	[118]
*B. atrox*	*Trypanosoma cruzi*	11.3 µM (epimastigote), 0.44 µM (trypomastigote) (IC_50_, 24 h), batroxicidin (cathelicidin)	[119]
*B. brazili*	*Candida albicans*, *Leishmania braziliensis* and *L. amazonensis*	120 µg/mL of Asp49-PLA_2_ and Lys49-PLA_2_ (50% of fungal inhibition, and 80–90% *Leishmania* inhibition)	[89]
*B. marajoensis*	*Leishmania amazonensis*, *L. chagasi promastigotes*	2.55–2.86 µg/mL (IC_50_), LAAO	[72]
*B. moojeni*	*L. amazonensis*, *L. chagasi*, *l. panamensisi*	1.08–1.44 µg/mL (EC_50_), LAAO	[120]
*B. moojeni*	*Trypanossoma cruzi*	8 µg/mL (inhibit 60% of *T. cruzi* growth), LAAO	[74]
*B. paoloensis*	*L. amazonensis*, *L. donovani*, *L. braziliensis*, *L. major*	1.03–1.59 µg/mL (IC_50_), LAAO	[75]
*B. pirajai*	*L. donovani*, *L. braziliensis*, *L. amazonensis*, *L. major*	5 µg/mL (inhibit <90% of *Leishmania* growth), LAAO	[76]
*Bungarus fasciatus*	*Aspergillus terreus*, *A. niculans*, *Chaetomium globosun*, *Candida albicans*, *Pichia pastoris*	0.3–18.7 µg/mL (MIC); Cath-BF (cathelicidin)	[121]
*Calloselasma rhodostoma*	*C. albicans*; *L. braziliensis*; *T. cruzi*	100 µg/mL (76% of *C. albicans* inhibition); 24.47 µg/mL (IC_50_ against *L. braziliensis*); 32 µg/mL (47% of *T. cruzi* killing), LAAO	[77]
*Crotalus durissus cascavella*	*Leishmania donovani*	2.39 µg/mL (IC_50_), LAAO	[78]
*C. d. cumamensis*	*Plasmodium falciparum*	0.6 µg/mL (IC_50_), Lys49-PLA_2_	[122]
*C. d. terrificus*	*Candida* spp, *Trichosporon* spp, and *Cryptococcus neoformans*	12.5-125 µg/mL (MIC)	[123]
*C. d. terrificus*	*Plasmodium falciparum*	1.87 µM (IC_50_), crotamine	[124]
*C. d. terrificus*	*Trypanosoma cruzi*	0.22–6.21 µM (EC_50_, against epimastigote and trypomastigote), Crotalicidin (cathelicidin); 9.5–33.1 (EC_50_, against trypomastigote), Crotalicidin fragments	[125]
*Hydrophis cyanocinctus*	*C. albicans*, *C. glabrata*, *Arcyria cinerea*	2.34–9.38 µg/mL (MIC), Hc-CATH(cathelicidin)	[126]
*Naja mossambica*	*Plasmodium falciparum*	2.3 pM (IC_50_), Group I PLA_2_	[127]
*N. scutatus*	*Plasmodium falciparum*	2.6 pM (IC_50_), Group I PLA_2_	[127]
*Sinonatrix annularis*	*C. albicans*, *C. glabrata*	18.75–37.5 µg/mL (MIC), SA-CATH	[128]
*Vipera ammodytes*	*Plasmodium falciparum*	2.8 pM (IC_50_), Lys49-PLA_2_	[127]

Microorganisms, microorganisms sensitive to antimicrobial activity; MIC, minimum inhibitory concentration; MBC, minimal bactericidal concentration; G−, Gram-negative bacteria; G+, Gram-positive bacteria; * the actual species name was consulted in the Reptile Database [19].

**Table 5:** Activity of snake cathelicidins on bacteria.

Snake	Microorganisms	Properties and Cathelicidin	Reference
*Bothrops atrox*	G−: *Escherichia coli*, *Pseudomonas aeruginosa*, *Klebsiella pneumoniae*; G+: *Enterococcus faecalis*, *Staphylococcus aureus*, *Acinetobacter baumanni*, *Streptococcus pyogenes*	0.25–128 µg/mL (MIC), Batroxicidin	[165]
*B. atrox*	G−: *K. pneumoniae*, *P. aeruginosa*, *E. coli*	8–16 µg/mL (MIC), 16–64 µg/mL (MBC), Batroxicidin	[166]
*Bungarus fasciatus*	G+: *B. subtilis*, *B. pumilus*, *Bacillus cereus*, *S. aureus*, *A. calcoaceticus*; G−: *E. coli*, *P. aeruginosa*, *Sphingobacterium siyangense*, *Sacharibacillus kuerlensis*, *Serratia marcescens*, *P. luteola*, *S. typhi*, *K. pneumoniae*; *Aspergillus terreus*, *A. niculans*, *Chaetomium globosum*, *Pichia pastoris*	0.3–100 µg/mL (MIC), resistant to 150 mM Na+, Cath-BF	[121]
*B. fasciatus*	G+: *Propionibacterium acnes*, *S. epidermidis*	1.2–4.7 µg/mL (MIC), anti-inflammatory activity in vivo, Cath-BF	[167]
*B. fasciatus*	G−: *E. coli*, *P. aeruginosa*; G+: *S. aureus*	2–32 µg/mL (MIC) protection against *P. aeruginosa* in infected burns, no resistance until the 8th subculture, Cath-BF	[11]
*B. fasciatus*	G−: *E. coli*, NDM-1-carrying *E. coli*; G+: *S. aureus*	4–36 µg/mL (MIC), 8–64 µg/mL (MBC), Cath-BF, Cbf-K16, Cbf-A7A13	[168]
*B. fasciatus*	*E. coli* (G−), *S. aureus* (G+)	4–16 µg/mL (MIC), local treatment in vivo is effective on vaginitis, Cath-BF	[169]
*B. fasciatus*	*E. coli* (G−)	BF-30 microspheres; antibacterial activity maintained during >15 days of release	[170]
*B. fasciatus*	*S. typhimurium* (G−)	attenuated the clinical symptoms in mice, Cath-BF	[171]
*B. fasciatus*	*P. aeruginosa* (G−)	innate immunity activation, pretreatment ameliorate pneumonia in vivo, Cath-BF	[172]
*B. fasciatus*	G−: *P. aeruginosa*, *E. coli*; G+: *A. baumannii*	8–64 µg/mL (MIC), Cath-BF recombinant and synthetic, active against *P. aeruginosa* and *A. baumannii* biofilms	[173]
*B. fasciatus*	G−: *P. aeruginosa*, *E. coli*; G+: *A. baumannii*, *MRSA*	8–128 µg/mL (MIC), active against *MRSA* biofilms, recombinant and synthetic Cath-BF	[126]
*Crotalus durissus terrificus*	G+: *E. faecalis*, *S. aureus*, *S. pyogenes*, *A. baumannii*; G−: *P. aeruginosa*, *K. pneumoniae*, *E. coli*	0.25–128 µg/mL (MIC), Crotalicidin	[165]
*C. d. terrificus*	G−: *P. aeruginosa*, *E. coli*, *K. pneumoniae*	2–16 µg/mL (MIC), 8–64 µg/mL (MBC), Crotalicidin	[166]
*Hydrophis cyanocinctus*	G−: *E. coli*, *Shigella dysenteriae*, *K. pneumoniae*, *K. oxytoca*, *Proteus mirabilis*, *Stenotrophomonas maltophilia*, *P. aeruginosa*, *S. paratyphi*, *Aeromonas sobria*, *A. hydrophila*, *A. veronni*, *Vibrio vulnificus*, *V. harveyi*, *V. fluvialis*, *V. alginolyticus*, *Edwardsiella tarda*; G+: *S. aureus*, *Bacillus cereus*, *B. subtilis*, *E. faecium*, *Nocardia asteroides*	2–75 µg/mL (MIC), Hc-CATH, Increased survival of mice to *P. aeruginosa* infection	[174]
*Naja atra*	*Burkholderia thailandesis* (G−)	3.66 µg/mL (EC50, 3 µg/mL inhibit biofilm formation), NA-CATH	[175]
*N. atra*	*Bacillus anthracis* (G+)	0.29 µg/mL (EC_50_), inactive against spores, 0.5 µg protect *Galleria mellonella* in vivo, NA-CATH	[176]
*N. atra*	*Francisella novicida* (G−)	1.54 µg/mL (EC_50_), NA-CATH	[177]
*N. atra*	G−: *E. coli*, *Aggregatibacter actinomycetemcomitas*	1.7 µg/mL (EC_50_), NA-CATH	[178]
*Ophiophagus hannah*	G−: *E. coli*, *P. aeruginosa*, *Enterobacter aerogenes*, *E. cloacae*	1–20 µg/mL (MIC), resistant to 1% NaCl, OH-CATH	[156]
*O. hannah*	G−: *E. coli*, *E. cloacae*, *E. aerogenes*, *P. aeruginosa*, *Haemophilus influenzae*, *K. pneumoniae*; G+: *S. aureus*, *E. faecalis*	1.56–25 µg/mL (MIC), resistant to serum, efficacious against *E. coli* bacteremia, OH-CATH	[179]
*O. hannah*	G−: *E. coli*, *P. aeruginosa*, *K. pneumoniae*, *MRSA*; G+: *E. faecalis*, *S. aureus*, *A. baumannii*, *S. pyogenes*	0.25–128 µg/mL (MIC), Oh-CRAMP	[165]
*O. hannah*	G+: *Acitenobacter sp.*, *Enterobacter sp.*, *Streptococcus pneumoniae*; G−: *Citrobacter sp.*, *Escherichia sp.*, *Klebsiella sp.*, *Proteus sp.*, *Pseudomonas sp.*, *Salmonella sp.*, *Serratia sp.*, *Stenotrophomonas maltophilia*, *Yersinia sp.*	8–64 µg/mL (MIC_90_), OH-CATH and D-OH-CATH	[180]
*O. hannah*	*P. aeruginosa* (G−)	3.25 µM (MIC), 5.07 µM (MIC after exposition to lung proteases), SnE1=OH-CATH	[181]
*Pseudonaja textilis*	G−: *E. coli*, *P. aeruginosa*, *K. pneumoniae*; G+: *E. faecalis*, *S. aureus*, *A. baumannii*, *S. pyogenes*	2–64 µg/mL (MIC), Pt_CRAMP	[165]
*Python bivittatus*	G−: *E. coli*, *P. aeruginosa*, *S. tiphimurium*; G+: *B. cereus*	1.5–46 µg/mL (MIC), Pb-CATH	[182]
*P. bivittatus*	G−: *Dysentery bacillus*, *E. coli*, *K. oxytoca*, *K. pneumoniae*, *S. paratyphi*, *P. aeruginosa*; G+: *Nocardia asteroids*, *S. aureus*, *B. cereus*, *E. faecalis*, *E. faecium*	1.17–75 µg/mL (MIC), 37.47–75.31% biofilm eradication using 2–5 MICs, CATHPb1, protection against MRSA and VRSA in vivo	[183]
*Trimerodytes annularis* (actual of *Sinonatrix annularis*) *	G−: *E. coli*, *K. pneumoniae*, *Shigela dysenteriae*, *P. aeruginosa*; G+: *S. aureus*, *B. cereus*, *B. subtillis*, *E. faecium*, *N. asteroids*	4.69–75 µg/mL (MIC), inhibit *E. coli* biofilm formation from 3.87% to 40.33% (1.25–40 µg/mL), SA-CATH	[127]

Microorganisms, microorganisms sensitive to antimicrobial activity; MIC, minimum inhibitory concentration; MBC, minimal bactericidal concentration; G−, Gram-negative bacteria; G+, Gram-positive bacteria; * the actual species name was consulted in Reptile Database [19]; NDM-1, New Delhi metallo-beta-lactamase-1; MRSA, methicillin-resistant *S. aureus*; VRSA, vancomycin-resistant *S. aureus*.

**Table 6:** Fragments derived from toxins and AMP and their antimicrobial action.

Snake	Microorganisms	Fragment or Peptide Name and Properties	Reference
*Agkistrodon contortrix laticinctus*	*Leishmania amazonensis*, *L. infantum chagasi*	Derived from Lys49-PLA_2_: **pACl**, 50.98–220.32 µM (EC_50_); **pAClR7**, 27.19–70.71 µM (EC_50_); derived from Lys49-PLA_2_	[190]
*A. piscivorus piscivorus*, *A. c. laticinctus*	G−: *P. aeruginosa*; G+: *S. aureus*; cancer cell lines: RAMOS, K562, NB4, and CEM cells	Derived from Lys49-PLA_2_ toxins (**p-AppK** and **p-Acl**)	[191]
*Bothrops asper*	G−: *Escherichia coli*, *Pasteurella multocida*, *Salmonella montevideo*, *S. typhi*, *Shigella sonnei*; G+: *Listeria monocytogenes*, *Staphylococcus aureus*, *Streptococcus pyogenes*, *Vibrio cholerae*	Derived from Lys49-PLA_2_: **p115-129**, 20–90 µg (MIC)	[192]
*B. asper*	*E. coli* (G−)	Derived from Lys49-PLA_2_: **p115-129**, 0.1–1 µM (MBC)	[97]
*B. asper*	G−: *S. aureus*, *S. thyphimurium*	Derived from Lys49-PLA_2_: **pEM1-10**, at 50 µg/mL (10–100% growth inhibition)	[97]
*B. asper*	G−: *Pseudomonas aeruginosa*, *Vibrio cholerae*, *Shigella sonnei*, *E. coli*, *S. typhimurium*, *Klebsiella pneumoniae*, *Brucella abortus*; G+: *Enterococcus faecalis*, *S. aureus*	Derived from myotoxin II, Lys49-PLA_2_: **pEM-2**, 1–250 µg/mL (MBC), at 100 µg/mL (25% protection of mice against *E. coli* and *S. enterica* peritoniti)	[97]
*B. brazili*	*E. coli* (G−); *Candida albicans*	**pep115-129 MTX-I** (derived from Asp 49 PLA_2_) and **pep115-129 MTX-II** (derived from Lys49-PLA_2_), at 120 µg/mL (<60% growth inhibition	[89]
*B. jararacussu*	*E. coli* (G−), *S. aureus* (G+)	Derived from Bothropstoxin I, Lys49-PLA_2_: **p-BthTX-I and (p-BthTX-I)_2_**, 4–128 µM (MIC)	[193]
*B. jararacussu*	G−: *E. coli*, G+: *S. aureus*, *S. epidermidis*, *E. faecium*	Derived from Bothropstoxin I, Lys49-PLA_2_: **p-BthTX-I and (p-BthTX-I)_2_**, 4–512 µM (MIC), eradication of *S. epidermidis* biofilm	[194]
*B. mattogrossensis*	*M. luteus* (G+)	**PS2** (derived from DefbBm02) and **PS4** (derived from DefbBm03), 26.1–26.6 µM (MIC)	[142]
*B. mattogrossensis*	G+: *Bacillus subtilis*, *S. aureus*, *Streptococcus pyogenes*; G−: *E. coli*, *K. pneumoniae*, *P. aeruginosa*, *S. typhimurium*	**BmLAO-f1, BmLAO-f2, BmLAO-f3** (derived from BmLAO, LAAO), 32–256 µg/mL (MIC)	[73]
*B. moojeni*	G+: S. aureus	Derived from Asp49-PLA_2_, **pBmTxJ**, 37.5 µM (MIC)	[195]
*Bungarus fasciatus*	G+: *B. subtilis*, *S. aureus*; G−: *E. coli*, *P. aeruginosa*, *Sacharibacillus kuerlensis*, *S. typhi*, *K. pneumoniae*; *C. albicans*, *Pichia pastoris*	**BF-15** (derived from cathelicidin Cath-BF), 1.2–75 µg/mL (MIC)	[121]
*B. fasciatus*	G−: *E. coli*, *S. aureus*, *P. aeruginosa*, *S. typhi*; G+: *B. subtilis*, *Enterobacter cloacae*; *C. albicans*	Fragments of BF-30 (Cath-BF), 1–128 µg/mL (MIC),	[196]
*B. fasciatus*	G+: *MRSA*, *S. aureus*, *S. epidermidis*; G−: *E. coli*	**Cbf-K16** (derived from Cath-BF), 4–64 µg/mL (MIC), synergism with ceftazidime/ampicilin in vivo	[197]
*B. fasciatus*	G−: *E. coli*; G+: *S. aureus*, *B. subtilis*; *C. albicans*	**ZY13** (derived from Cath-BF), 0.59–75 µg/mL (MIC), resistant to 150 mM NaCl	[198]
*B. fasciatus*	G−: *E. coli*, *K. pneumoniae*, *P. aeruginosa*; G+: *S. aureus*, *S. epidermidis*	**Cbf-14**, derived from Cath BF, 8–64 µg/mL (MIC), 16–128 µg/mL (MBC), anti-inflammatory activity, protection in vivo	[199]
*B. fasciatus*	G−: *E. coli*, *K. pneumoniae*; G+: *MRSA*	**Cath-A** and **Cath-B**, derived from Cath-BF	[200]
*Cerrophidion. godmani* *	G−: *E. coli*; *Leishmania braziliensis*, *L. amazonensis promastigotas*	Derived from Asp49-PLA_2_, **pCergo**, 75 µM (MIC); 93.69–110.40 µM (EC_50_)	[195]
*Crotalus durissus terrificus*	G+: *E. faecalis*, *S. aureus*; G−: *S. pyogenes*, *P. aeruginosa*, *K. pneumoniae*, *E. coli*, *A. baumannii*	**Ctn(1–14)**, **Ctn(15–34),** (derived from Crotalicidin, cathelicidin), 0.25–128 µg/mL (MIC)	[201]
*C. d. terrificus*	*Candida krusei*, *C. glabrata*, *parapsilosis*, *C. tropicalis*, *C. guilliermondii*, *Trichosporon spp.*	**C1, C2**, derived from crotamine, 2.5–40 µM (MIC), substituição de C por S diminui a atividade	[202]
*C. d. terrificus*	*Candida parapsilosis*, *C. krusei*, *C. tropicalis*, *C. albicans*, *Cryptococcus laurenti*, *Microsporum canis*	**Ctn(15–34),** derived from Crotalicidin, 5–20 µM (MIC),	[203]
*C. d. terrificus*	G−: *E. coli*, *S. typhi*, *Xanthomonas oryzae*, *X. axonopodis*, *Vibrio cholerae*; G+: *B. cereus*, *MRSA*; *C. albicans*, *Fusarium solani*	**CyLoP-1**, derived from crotamine, 5–40 µM (MIC)	[204]
*C. d. terrificus*	*C. albicans*	**Ctn(15–34)** Crotalicidin, synergism with amphotericin B	[205]
*C. d. terrificus*	G−: *E. coli*, *P. aeruginosa*	**Ctn(15–34)** Crotalicidin, 3.13–12.5 µM (MIC), 6.25–50 µM (MBC)	[203]
*C. d. terrificus*	*P. aeruginosa* (G−)	**SnV1**, derived from Crotalicidin, 0.877–5.42 µM (MIC) before and after treatment with lung proteases	[181]
*C. d. terrificus*	G−: *E. coli*, *Citrobacter freundii*; G+: *S. aureus*, *M. luteus.*	**PS1** and **PS6**, derived from crotamine, 28.4–56.5 µM (MIC)	[142]
*C. d. terrificus*	G+: *Mycobacterium smegmatis*, *M. fortuitum*, *M. wolinskyi*	**CyLoP-1**, derived from crotamine, 10–20 µM (MIC), 10–20 µM (MBC)	[206]
*C. oreganus abyssus*	G−: *E. coli*, *P. aeruginosa*; G+: *S. aureus*	**pC-CoaTxII** (C-terminal fragment 115–129 from Lys49-PLA_2_), inhibited <90% of bacterial growth at 5.95 µM.	[207]
*Hydrophis cyanocinctus*	*P. aeruginosa* (G−)	**Sn1, Sn1A, Sn1b**, derived from Hc-CATH, 0.66–45.6 µM (MIC) before and after treatment with lung proteases	[181]
*Lachesis muta*	G−: *E. coli*, *K. pneumoniae*, *C. freundii*; G+: *S. aureus*, *M. luteus.*	**PS3** and **PS5**, derived from DefbLm02, 13.9–110 µM (MIC)	[142]
*Naja atra*	G−: *E. coli*, *Aggregatibacter actinomycetemcomitans*	**ATRA-1** and **ATRA-1A**, fragments of CATH-ATRA, 0.88–160 µg/mL (EC_50_)	[178]
*N. atra*	*Francisella novicida* (G−)	**ATRA-1** and **ATRA-1A**, fragments of CATH-ATRA, 8.95–147.9 µg/mL (EC_50_)	[177]
*N. atra*	*S. aureus* (G+)	**ATRA-1** and **ATRA-1A**, fragments of CATH-ATRA, 0.52–18 µg/mL (EC_50_), inhibit biofilm formation	[208]
*N. atra*	G−: *E. coli*, *P. aeruginosa*; G+: *B. cereus*, *S. aureus.*	**ATRA-1** and **ATRA-1A**, derived from NA-CATH, 1.9–72.9 µg/mL (EC_50_)	[209]
*N. atra*	*Burkholderia thailandensis* (G−)	**ATRA-1A**, derived from NA-CATH, 7–14 µg/mL (EC_50_)	[175]
*N. atra*	G−: *E. coli*, *P. aeruginosa*, *K. pneumoniae*, *E. cloacae*, *B. cepacia*, *P. mirabilis*, *Moraxella catarrhalis*; G+: *A. baumannii*, *S. aureus*, *E. hirae*, *S. agalactiae*; *Candida albicans*, *C. glabrata*, *Malassezia pachydermatis*, *Mycobacterium smegmatis*, *M. fortuitum*	**NP-0, NP-2, NCP-3, NCP-3a, NCP-3b,** derived from cardiotoxin, 1.6–50 µg/mL (MBC), active at 250 mM NaCl	[210]
*Ophiophagus hannah*	G−: *E. coli*, *P. aeruginosa*, *Enterobacter aerogenes*, *E. cloacae*; G+: *S. aureus*,	**Fragments (3–34), (5–34), (1–24) and (3–17)**, derived from OH-CATH, 4–24 µg/mL (MIC)]	[211]
*O. hannah*	G−: *E. coli*, *E. cloacae*, *E. aerogenes*, *P. aeruginosa*, *H. influenzae*, *K. pneumoniae*; G+: *S. aureus*, *E. faecalis*	**OH-CM6,** derived from OH-CATH-30, 1.56–25 µg/mL (MIC), active against *E. coli* bacteremia in vivo, stable in serum	[187]
*O. hannah*	*P. aeruginosa* (G−)	**SnE1**, derived from OH-CATH, 3.25–5.07 µM (MIC before and after treatment with lung proteases)	[181]

Microorganisms, microorganisms sensitive to antimicrobial activity; MIC, minimum inhibitory concentration; MBC, minimal bactericidal concentration; G−, Gram-negative bacteria; G+, Gram-positive bacteria; * the actual species name was consulted in the Reptile Database [19].

## Data Availability

β-defensin sequences of *Sistrurus miliarius* can be retrieved at GenBank, accession numbers MT024631 to MT024638.

## Supplementary Materials

The following supporting information can be downloaded at: https://www.mdpi.com/article/10.3390/ani13040744/s1, Table S1: Characteristics of snake cathelicidins. The sequences were obtained from the literature or NCBI databases. The net charge was calculated using the Henderson-Hasselbalch equation and the Lehninger pKa Scale; Table S2: Snake cathelicidins genome position. The contigs containing cathelicidin genes were identified through recursive BLAST searches of the WGS NCBI database, and the approximate positions of the genes were recorded. The sequences represented in this table are all partial and have not been previously reported in the literature. In silico approach has been used to search the sequences, realize the alignments, and generate the Phylogenetic tree [153,218,219,220,221,222,223,224,225,226].

## References

[1] Zasloff M. (2002). Antimicrobial peptides of multicellular organisms. Nature.

[2] Nunes L.G.P., Reichert T., Machini M.T. (2021). His-Rich Peptides, Gly- and His-Rich Peptides: Functionally Versatile Compounds with Potential Multi-Purpose Applications. Int. J. Pept. Res. Ther..

[3] Santana F.L., Estrada K., Ortiz E., Corzo G. (2021). Reptilian β-defensins: Expanding the repertoire of known crocodylian peptides. Peptides.

[4] Pyron R.A., Burbrink F.T., Wiens J.J. (2013). A phylogeny and revised classification of Squamata, including 4161 species of lizards and snakes. BMC Evol. Biol..

[5] Reeks T.A., Fry B.G., Alewood P.F. (2015). Privileged frameworks from snake venom. Cell. Mol. Life Sci..

[6] Samy R.P., Gopalakrishnakone P., Satyanarayanajois S.D., Stiles B.G., Chow V.T.K. (2013). Snake Venom Proteins and Peptides as Novel Antibiotics against Microbial Infections. Curr. Proteom..

[7] Assis R.A., Bittar B.B., Amorim N.P.L., Carrasco G.H., Silveira E.D.R., Benvindo-Souza M., Santos L.R.S. (2022). Studies about Snake Peptides: A Review about Brazilian Contribution. Braz. Arch. Biol. Technol..

[8] Hancock R.E.W. (1997). Peptide antibiotics. Lancet.

[9] Carvalho L.A.C., Remuzgo C., Perez K.R., Machini M.T. (2015). Hb40-61a: Novel analogues help expanding the knowledge on chemistry, properties and candidacidal action of this bovine α-hemoglobin-derived peptide. Biochim. Biophys. Acta.

[10] Radzishevsky I.S., Rotem S., Bourdetsky D., Navon-Venezia S., Carmeli Y., Mor A. (2007). Improved antimicrobial peptides based on acyl-lysine oligomers. Nat. Biotechnol..

[11] Zhou H., Dou J., Wang J., Chen L., Wang H., Zhou W., Li Y., Zhou C. (2011). The antibacterial activity of BF-30 in vitro and in infected burned rats is through interference with cytoplasmic membrane integrity. Peptides.

[12] Xie J.P., Yue J., Xiong Y.L., Wang W.Y., Yu S.Q., Wang H.H. (2003). In vitro activities of small peptides from snake venom against clinical isolates of drug-resistant *Mycobacterium tuberculosis*. Int. J. Antimicrob. Agents.

[13] Zimmerman L.M., Vogel L.A., Bowden R.M. (2010). Understanding the vertebrate immune system: Insights from the reptilian perspective. J. Exp. Biol..

[14] Rios F.M., Zimmerman L.M. (2015). Immunology of Reptiles. eLS.

[15] Grego K.F., Alves J.A.S., Albuquerque L.C.R., Fernandes W. (2006). Referências hematológicas para a jararaca de rabo branco (*Bothrops leucurus*) recém capturadas da natureza. Arq. Bras. Med. Vet. Zootec..

[16] Carvalho M.P.N., Queiroz-Hazarbassanov N.G.T., Massoco C.O., Sant’Anna S.S., Lourenço M.M., Levin G., Sogayar M.C., Grego K.F., Catão-Dias J.L. (2017). Functional characterization of neotropical snakes peripheral blood leukocytes subsets: Linking flow cytometry cell features, microscopy images and serum corticosterone levels. Dev. Comp. Immunol..

[17] Farag M.A., El Ridi R. (1986). Proliferative Responses of Snake Lymphocytes to Concanavalin A. Dev. Comp. Immunol..

[18] Saad A.H. (1989). Sex-Associated Differences in the Mitogenic Responsiveness of Snake Blood Lymphocytes. Dev. Comp. Immunol..

[19] Uetz P., Freed P., Aguilar R., Hošek J. (2022). The Reptile Database. https://www.reptile-database.org.

[20] Vogel C.W., Muller-Eberhard J. (1985). The Cobra Complement System: I. The Alternative Pathway of Activation. Dev. Comp. Immunol..

[21] Graham S.P., Fielman K.T., Mendonça M.T. (2017). Thermal performance and acclimatization of a component of snake (*Agkistrodon piscivorus*) innate immunity. J. Exp. Zool..

[22] Bals R., Wilson J.M. (2003). Cathelicidins—A family of multifunctional antimicrobial peptides. Cell. Mol. Life Sci..

[23] Graham S.P., Earley R.L., Guyer C., Mendonça M.T. (2011). Innate immune performance and steroid hormone profiles of pregnant versus nonpregnant cottonmouth snakes (*Agkistrodon piscivorus*). Gen. Comp. Endocr..

[24] French S.S., Neuman-Lee L.A. (2012). Improved ex vivo method for microbiocidal activity across vertebrate species. Biol. Open.

[25] Figueiredo A.C., Nogueira L.A.K., Titon S.C.M., Gomes F.R., Carvalho J.E. (2022). Immune and hormonal regulation of the *Boa constrictor* (Serpentes; Boidae) in response to feeding. Comp. Biochem. Physiol. A.

[26] Brusch G.A., Mills A.M., Walman R.M., Masuda G., Byeon A., DeNardo D.F., Stahlschmidt Z.R. (2020). Dehydration enhances cellular and humoral immunity in a mesic snake community. J. Exp. Zool..

[27] Brusch G.A., DeNardo D.F. (2019). Egg desiccation leads to dehydration and enhanced innate immunity in python embryos. Dev. Comp. Immunol..

[28] Brusch G.A., DeNardo D.F. (2017). When less means more: Dehydration improves innate immunity in rattlesnakes. J. Exp. Biol..

[29] Fabrıcio-Neto A., Madelaire C.B., Gomes F.R., Andrade D.V. (2019). Exposure to fluctuating temperatures leads to reduced immunity and to stress response in rattlesnakes. J. Exp. Biol..

[30] Baker S.J., Merchant M.E. (2018). Antibacterial properties of plasma from the prairie rattlesnake (*Crotalus viridis*). Dev. Comp. Immunol..

[31] Brusch G.A., Christian K., Brown G.P., Shine R., DeNardo D.F. (2019). Dehydration enhances innate immunity in a semiaquatic snake from the wet-dry tropics. J. Exp. Zool..

[32] Tripathi M.K., Singh R. (2014). Differential Suppressive Effects of Testosterone on Immune Function in Fresh Water Snake, *Natrix piscator*: An In Vitro Study. PLoS ONE.

[33] Tripathi M.K., Singh R., Pati A.K. (2015). Daily and Seasonal Rhythms in Immune Responses of Splenocytes in the Freshwater Snake, *Natrix piscator*. PLoS ONE.

[34] Singh A., Singh R., Tripathi M.K. (2020). Photoperiodic manipulation modulates the innate and cell mediated immune functions in the freshwater snake, *Natrix piscator*. Sci. Rep..

[35] Luoma R.L., Butler M.W., Stahlschmidt Z.R. (2016). Plasticity of immunity in response to eating. J. Exp. Biol..

[36] Lind C.M., Agugliaro J., Farrell T.M. (2020). The metabolic response to an immune challenge in a viviparous snake, *Sistrurus miliarius*. J. Exp. Biol..

[37] McCoy C.M., Lind C.M., Farrell T.M. (2017). Environmental and physiological correlates of the severity of clinical signs of snake fungal disease in a population of pigmy rattlesnakes, *Sistrurus miliarius*. Conserv. Physiol..

[38] Sparkman A.M., Palacios M.G. (2009). A test of life-history theories of immune defence in two ecotypes of the garter snake, *Thamnophis elegans*. J. Anim. Ecol..

[39] Palacios M.G., Sparkman A.M., Bronikowski A.M. (2011). Developmental plasticity of immune defence in two life-history ecotypes of the garter snake, *Thamnophis elegans*—A common-environment experiment. J. Anim. Ecol..

[40] Neuman-Lee L.A., Fokidis H.B., Spence A.R., van der Walt M., Smith G.D., Durham S., Smith S.S. (2015). Food restriction and chronic stress alter energy use and affect immunity in an infrequent feeder. Funct. Ecol..

[41] Palacios M.G., Bronikowski A.M. (2017). Immune variation during pregnancy suggests immune component-specific costs of reproduction in a viviparous snake with disparate life-history strategies. J. Exp. Zool..

[42] Palacios M.G., Gangloff E.J., Reding D.M., Bronikowski A.M. (2020). Genetic background and thermal environment differentially influence the ontogeny of immune components during early life in an ectothermic vertebrate. J. Anim. Ecol..

[43] Spence A.R., French S.S., Hopkins G.R., Durso A.M., Hudson S.B., Smith G.D., Neuman-Lee L.A. (2020). Long-term monitoring of two snake species reveals immune–endocrine interactions and the importance of ecological context. J. Exp. Zool..

[44] Combrink L.L., Bronikowski A.M., Miller D.A.W., Sparkman A.M. (2021). Current and time-lagged effects of climate on innate immunity in two sympatric snake species. Ecol. Evol..

[45] Neuman-Lee L.A., van Wettere A.J., French S.S. (2019). Interrelations among Multiple Metrics of Immune and Physiological Function in a Squamate, the Common Gartersnake (*Thamnophis sirtalis*). Physiol. Biochem. Zool..

[46] Kobolkuti L., Cadar D., Czirjak G., Niculae M., Kiss T., Sandru C., Spinu M. (2012). The Effects of Environment and Physiological Cyclicity on the Immune System of *Viperinae*. Sci. World J..

[47] Stiles B.G., Sexton F.W., Weinstein S.A. (1991). Antibacterial Effects of Different Snake Venoms: Purification and Characterization of Antibacterial Proteins from *Pseudechis australis* (Australian King Brown or Muga Snake) Venom. Toxicon.

[48] Skarnes R.C. (1970). L-Amino-acid Oxidase, a Bactericidal System. Nature.

[49] Sulca-Lopez M.A., Remuzgo C., Cardenas J., Kiyota S., Cheng E., Bemquerer M.P., Machini M.T. (2017). Venom of the Peruvian snake *Bothriopsis oligolepis*: Detection of antibacterial activity and involvement of proteolytic enzymes and C-type lectins in growth inhibition of *Staphylococcus aureus*. Toxicon.

[50] Rheubert J.L., Meyer M.F., Strobel R.M., Pasternak M.A., Charvat R.A. (2020). Predicting antibacterial activity from snake venom proteomes. PLoS ONE.

[51] Arlinghaus F.T., Eble J.A. (2012). C-type lectin-like proteins from snake venoms. Toxicon.

[52] Murakami M.T., Zela S.P., Gava L.M., Michelan-Duarte S., Cintra A.C.O., Arni R.K. (2003). Crystal structure of the platelet activator convulxin, a disulfide-linked a4b4 cyclic tetramer from the venom of *Crotalus durissus terrificus*. Biochem. Biophys. Res. Commun..

[53] Rádis-Baptista G., Moreno F.B.M.B., Nogueira L.L., Martins A.M.C., Toyama D.O., Toyama M.H., Cavada B.S., Azevedo W.F., Yamane T. (2006). Crotacetin, a Novel Snake Venom C-Type Lectin Homolog of Convulxin, Exhibits an Unpredictable Antimicrobial Activity. Cell Biochem. Biophys..

[54] Castanheira L.E., Nunes D.C.O., Cardoso T.M., Santos P.S., Goulart L.R., Rodrigues R.S., Richardson M., Borges M.H., Yoneyama K.A.G., Rodrigues V.M. (2013). Biochemical and functional characterization of a C-type lectin (BpLec) from *Bothrops pauloensis* snake venom. Int. J. Biol. Macromol..

[55] Nunes E.S., Souza M.A.A., Vaz A.F.M., Santana G.M.S., Gomes F.S., Coelho L.C.B.B., Paiva P.M.G., Silva R.M.L., Silva-Lucca R.A., Oliva M.L.V. (2011). Purification of a lectin with antibacterial activity from *Bothrops leucurus* snake venom. Comp. Biochem. Physiol. B.

[56] Klein R.C., Fabres-Klein M.H., de Oliveira L.L., Feio R.N., Malouin F., Ribon A.O.B. (2015). A C-Type Lectin from *Bothrops jararacussu* Venom Disrupts Staphylococcal Biofilms. PLoS ONE.

[57] Moura-da-Silva A.M., Theakston R.D.G., Crampton J.M. (1996). Evolution of Disintegrin Cysteine-Rich and Mammalian Matrix-Degrading Metalloproteinases: Gene Duplication and Divergence of a Common Ancestor Rather than Convergent Evolution. J. Mol. Evol..

[58] Bazaa A., Juárez P., Marrakchi N., Lasfer Z.B., El Ayeb M., Harrison R.A., Calvete J.J., Sanz L. (2007). Loss of Introns Along the Evolutionary Diversification Pathway of Snake Venom Disintegrins Evidenced by Sequence Analysis of Genomic DNA from *Macrovipera lebetina transmediterranea* and *Echis ocellatus*. J. Mol. Evol..

[59] Samy R.P., Gopalakrishnakone P., Chow V.T.K., Ho B. (2008). Viper Metalloproteinase (*Agkistrodon halys* Pallas) with Antimicrobial Activity against Multi-Drug Resistant Human Pathogens. J. Cell. Physiol..

[60] Allane D., Oussedik-Oumehdi H., Harrat Z., Seve M., Laraba-Djebari F. (2018). Isolation and characterization of an anti-leishmanial disintegrin from *Cerastes cerastes* venom. J. Biochem. Mol. Toxicol..

[61] Serrano S.M.T., Maroun R.C. (2005). Snake venom serine proteinases: Sequence homology vs. substrate specificity, a paradox to be solved. Toxicon.

[62] Castro H.C., Zingali R.B., Albuquerque M.G., Pujol-Luz M., Rodrigues C.R. (2004). Snake venom thrombin-like enzymes: From reptilase to now. Cell. Mol. Life Sci..

[63] Ali S.A., Stoeva S., Abbasi A., Alam J.M., Kayed R., Faigle M., Neumeister B., Voelter W. (2000). Isolation, Structural, and Functional Characterization of an Apoptosis-Inducing L-Amino Acid Oxidase from Leaf-Nosed Viper (*Eristocophis macmahoni*) Snake Venom. Arch. Biochem. Biophys..

[64] Du X.-Y., Clemetson K.J. (2002). Snake venom L-amino acid oxidases. Toxicon.

[65] Takatsuka H., Sakurai Y., Yoshioka A., Kokubo T., Usami Y., Suzuki M., Matsui T., Titani K., Yagi H., Matsumoto M. (2001). Molecular characterization of L-amino acid oxidase from *Agkistrodon halys blomhoffii* with special reference to platelet aggregation. Biochim. Biophys. Acta.

[66] Kasai K., Nakano M., Ohishi M., Nakamura T., Miura T. (2021). Antimicrobial properties of L-amino acid oxidase: Biochemical features and biomedical applications. Appl. Microbiol. Biotechnol..

[67] Sun M.-Z., Guo C., Tian Y., Chen D., Greenaway F.T., Liu S. (2010). Biochemical, functional and structural characterization of Akbu-LAAO: A novel snake venom L-amino acid oxidase from *Agkistrodon blomhoffii ussurensis*. Biochimie.

[68] Zhang H., Yang Q., Sun M., Teng M., Niu L. (2004). Hydrogen Peroxide produced by Two Amino Acid Oxidases Mediates Antibacterial Actions. J. Microbiol..

[69] Muñoz L.J.V., Estrada-Gomez S., Núñez V., Sanz L., Calvete J.J. (2014). Characterization and cDNA sequence of *Bothriechis schlegelii* L-aminoacid oxidase with antibacterial activity. Int. J. Biol. Macromol..

[70] Stábeli R.G., Marcussi S., Carlos G.B., Pietro R.C.L.R., Selistre-de-Araújo H.S., Giglio J.R., Oliveira E.B., Soares A.M. (2004). Platelet aggregation and antibacterial effects of an L-amino acid oxidase purified from *Bothrops alternatus* snake venom. Bioorg. Med. Chem..

[71] Ciscotto P., Avila R.A.M., Coelho E.A.F., Oliveira J., Diniz C.G., Farías L.M., Carvalho M.A.R., Maria W.S., Sanchez E.F., Borges A. (2009). Antigenic, microbicidal and antiparasitic properties of an L-amino acid oxidase isolated from *Bothrops jararaca* snake venom. Toxicon.

[72] Torres A.F.C., Dantas R.T., Toyama M.H., Diz Filho E., Zara F.J., Queiroz M.G.R., Nogueira N.A.P., Oliveira M.R., Toyama D.O., Monteiro H.S.A. (2010). Antibacterial and antiparasitic effects of *Bothrops marajoensis* venom and its fractions: Phospholipase A_2_ and L-amino acid oxidase. Toxicon.

[73] Okubo B.M., Silva O.N., Migliolo L., Gomes D.G., Porto W.F., Batista C.L., Ramos C.S., Holanda H.H.S., Dias S.C., Franco O.L. (2012). Evaluation of an Antimicrobial L-Amino Acid Oxidase and Peptide Derivatives from *Bothropoides mattogrosensis* Pitviper Venom. PLoS ONE.

[74] Stábeli R.G., Sant’Ana C.D., Ribeiro P.H., Costa T.R., Ticli F.K., Pires M.G., Nomizo A., Albuquerque S., Malta-Neto N.R., Marins M. (2007). Cytotoxic L-amino acid oxidase from *Bothrops moojeni*: Biochemical and functional characterization. Int. J. Biol. Macromol..

[75] Rodrigues R.S., Silva J.F., França J.B., Fonseca F.P.P., Otaviano A.R., Silva F.H., Hamaguchi A., Magro A.J., Braz A.S.K., Santos J.I. (2009). Structural and functional properties of Bp-LAAO, a new L-amino acid oxidase isolated from *Bothrops pauloensis* snake venom. Biochimie.

[76] Izidoro L.F.M., Ribeiro M.C., Souza G.R.L., Sant’Ana C.D., Hamaguchi A., Homsi-Brandeburgo M.I., Goulart L.R., Beleboni R.O., Nomizo A., Sampaio S.V. (2006). Biochemical and functional characterization of an L-amino acid oxidase isolated from *Bothrops pirajai* snake venom. Bioorg. Med. Chem..

[77] Costa T.R., Menaldo D.L., Silva C.P., Sorrechia R., Albuquerque S., Pietro R.C.L.R., Ghisla S., Antunes L.M.G., Sampaio S.V. (2015). Evaluating the microbicidal, antiparasitic and antitumor effects of CR-LAAO from *Calloselasma rhodostoma* venom. Int. J. Biol. Macromol..

[78] Toyama M.H., Toyama D.O., Passero L.F.D., Laurenti M.D., Corbett C.E., Tomokane T.Y., Fonseca F.V., Antunes E., Joazeiro P.P., Beriam L.O.S. (2006). Isolation of a new L-amino acid oxidase from *Crotalus durissus cascavella* venom. Toxicon.

[79] Vargas L.J., Quintana J.C., Pereañez J.A., Núñez V., Sanz L., Calvete J. (2013). Cloning and characterization of an antibacterial L-amino acid oxidase from *Crotalus durissus cumanensis* venom. Toxicon.

[80] Zhong S.-R., Jin Y., Wu J.-B., Jia Y.-H., Xu G.-L., Wang G.-C., Xiong Y.-L., Lu Q.-M. (2009). Purification and characterization of a new L-amino acid oxidase from *Daboia russellii siamensis* venom. Toxicon.

[81] Samel M., Tonismagi K., Ronnholm G., Vija H., Siigur J., Kalkkinen N., Siigur E. (2008). L-Amino acid oxidase from *Naja naja oxiana* venom. Comp. Biochem. Physiol. B.

[82] Lee M.L., Tan N.H., Fung S.Y., Sekaran S.D. (2011). Antibacterial action of a heat-stable form of L-amino acid oxidase isolated from king cobra (*Ophiophagus hannah*) venom. Comp. Biochem. Physiol. C.

[83] Phua C.S., Vejayan J., Ambu S., Ponnudurai G., Gorajana A. (2012). Purification and antibacterial activities of an L-amino acid oxidase from king cobra (*Ophiophagus hannah*) venom. J. Venom. Anim. Toxins Incl. Trop. Dis..

[84] Zhang Y.-J., Wang J.-H., Lee W.-H., Wang Q., Liu H., Zheng Y.-T., Zhang Y. (2003). Molecular characterization of *Trimeresurus stejnegeri* venom L-amino acid oxidase with potential anti-HIV activity. Biochem. Biophys. Res. Commun..

[85] Tõnismagi K., Samel M., Trummal K., Ronnholm G., Siigur J., Kalkkinen N., Siigur E. (2006). L-Amino acid oxidase from *Vipera lebetina* venom: Isolation, characterization, effects on platelets and bacteria. Toxicon.

[86] Arias S.P., Rey-Suárez P., Pereáñez J.A., Acosta C., Rojas M., Santos L.D., Ferreira R.S., Núñez V. (2017). Isolation and Functional Characterization of an Acidic Myotoxic Phospholipase A_2_ from Colombian *Bothrops asper* Venom. Toxins.

[87] Lomonte B. (2023). Lys49 myotoxins, secreted phospholipase A_2_-like proteins of viperid venoms: A comprehensive review. Toxicon.

[88] Vargas L.J., Londoño M., Quintana J.C., Rua C., Segura C., Lomonte B., Núñez V. (2012). An acidic phospholipase A_2_ with antibacterial activity from *Porthidium nasutum* snake venom. Comp. Biochem. Physiol. B Biochem. Mol. Biol..

[89] Costa T.R., Menaldo D.L., Oliveira C.Z., Santos-Filho N.A., Teixeira S.S., Nomizo A., Fuly A.L., Monteiro M.C., Souza B.M., Palma M.S. (2008). Myotoxic phospholipases A_2_ isolated from *Bothrops brazili* snake venom and synthetic peptides derived from their C-terminal region: Cytotoxic effect on microorganism and tumor cells. Peptides.

[90] Páramo L., Lomonte B., Pizarro-Cerdá J., Bengoechea J.A., Gorvel J.-P., Moreno E. (1998). Bactericidal activity of Lys49 and Asp49 myotoxic phospholipases A_2_ from *Bothrops asper* snake venom Synthetic Lys49 myotoxin II-(115−129)-peptide identifies its bactericidal region. Eur. J. Biochem..

[91] Muller V.D.M., Russo R.R., Cintra A.C.O., Sartim M.A., Alves-Paiva R.M., Figueiredo L.T.M., Sampaio S.V., Aquino V.H. (2012). Crotoxin and phospholipases A_2_ from *Crotalus durissus terrificus* showed antiviral activity against dengue and yellow fever viruses. Toxicon.

[92] Muller V.D., Soares R.O., dos Santos-Junior N.N., Trabuco A.C., Cintra A.C., Figueiredo L.T., Caliri A., Sampaio S.V., Aquino V.H. (2014). Phospholipase A_2_ Isolated from the Venom of *Crotalus durissus terrificus* Inactivates Dengue virus and Other Enveloped Viruses by Disrupting the Viral Envelope. PLoS ONE.

[93] Brenes H., Loría G.D., Lomonte B. (2020). Potent virucidal activity against Flaviviridae of a group IIA phospholipase A_2_ isolated from the venom of *Bothrops asper*. Biologicals.

[94] Fenard D., Lambeau G., Valentin E., Lefebvre J.-C., Lazdunski M., Doglio A. (1999). Secreted phospholipases A_2_, a new class of HIV inhibitors that block virus entry into host cells. J. Clin. Investig..

[95] Shimizu J.F., Pereira C.M., Bittar C., Batista M.N., Campos G.R.F., da Silva S., Cintra A.C.O., Zothner C., Harris M., Sampaio S.V. (2017). Multiple effects of toxins isolated from *Crotalus durissus terrificus* on the hepatitis C virus life cycle. PLoS ONE.

[96] Roberto P.G., Kashima S., Marcussi S., Pereira J.O., Astolfi-Filho S., Nomizo A., Giglio J.R., Fontes M.R.M., Soares A.M., França S.C. (2004). Cloning and Identification of a Complete cDNA Coding for a Bactericidal and Antitumoral Acidic Phospholipase A_2_ from *Bothrops jararacussu* Venom. Protein J..

[97] Santamaría C., Larios S., Ângulo Y., Pizarro-Cerda J., Gorvel J.-P., Moreno E., Lomonte B. (2005). Antimicrobial activity of myotoxic phospholipases A_2_ from crotalid snake venoms and synthetic peptide variants derived from their C-terminal region. Toxicon.

[98] Aragão E.A., Chioato L., Ward R.J. (2008). Permeabilization of *E. coli* K12 inner and outer membranes by bothropstoxin-I, A LYS49 phospholipase A_2_ from *Bothrops jararacussu*. Toxicon.

[99] Barbosa P.S.F., Martins A.M.C., Havt A., Toyama D.O., Evangelista J.S.A.M., Ferreira D.P.P., Joazeiro P.P., Beriam L.O.S., Toyama M.H., Fonteles M.C. (2005). Renal and antibacterial effects induced by myotoxin I and II isolated from *Bothrops jararacussu* venom. Toxicon.

[100] Corrêa E.A., Kayano A.M., Diniz-Sousa R., Setúbal S.S., Zanchi F.B., Zuliani J.P., Matos N.B., Almeida J.R., Resende L.M., Marangoni S. (2016). Isolation, structural and functional characterization of a new Lys49 phospholipase A_2_ homologue from *Bothrops neuwiedi urutu* with bactericidal potential. Toxicon.

[101] Xu C., Ma D., Yu H., Li Z., Liang J., Lin G., Zhang Y., Lai R. (2007). A bactericidal homodimeric phospholipases A_2_ from *Bungarus fasciatus* venom. Peptides.

[102] Wen Y.-L., Wu B.-J., Kao P.-H., Fu Y.-S., Chang L.-S. (2013). Antibacterial and membrane-damaging activities of β-bungarotoxin B chain. J. Pept. Sci..

[103] Samy R.P., Kandasamy M., Gopalakrishnakone P., Stiles B.G., Rowan E.G., Becker D., Shanmugam M.K., Sethi G., Chow V.T.K. (2014). Wound Healing Activity and Mechanisms of Action of an Antibacterial Protein from the Venom of the Eastern Diamondback Rattlesnake (*Crotalus adamanteus*). PLoS ONE.

[104] Toyama M.H., Toyama D.O., Joazeiro P.P., Carneiro E.M., Beriam L.O.S., Marangoni L.S., Boschero A.C. (2005). Biological and Structural Characterization of a New PLA_2_ from the *Crotalus durissus collilineatus* Venom. Protein J..

[105] Diz Filho E.B.S., Marangoni S., Toyama D.O., Fagundes F.H.R., Oliveira S.C.B., Fonseca F.V., Calgarotto A.K., Joazeiro P.P., Toyama M.H. (2009). Enzymatic and structural characterization of new PLA_2_ isoform isolated from white venom of *Crotalus durissus ruruima*. Toxicon.

[106] Samy R.P., Pachiappan A., Gopalakrishnakone P., Thwin M.M., Hian Y.E., Chow V.T.K., Bow H., Weng J.T. (2006). In vitro antimicrobial activity of natural toxins and animal venoms tested against *Burkholderia pseudomallei*. BMC Infect. Dis..

[107] Samy R.P., Gopalakrishnakone P., Thwin M.M., Chow T.K.V., Bow H., Yap E.H., Thong T.W.J. (2007). Antibacterial activity of snake, scorpion and bee venoms: A comparison with purified venom phospholipase A_2_ enzymes. J. Appl. Microbiol..

[108] Almeida J.R., Lancellotti M., Soares A.M., Calderon L.A., Ramírez D., González W., Marangoni S., da Silva S.L. (2016). CoaTx-II, a new dimeric Lys49 phospholipase A2 from *Crotalus oreganus abyssus* snake venom with bactericidal potential: Insights into its structure and biological roles. Toxicon.

[109] Sudarshan S., Dhananjaya B.L. (2014). Antibacterial Potential of a Basic Phospholipase A_2_ (VRV_PL_V) of *Daboia russellii pulchella* (Russell’s Viper) Venom. Biochemistry (Mosc.).

[110] Sudharshan S., Dhananjaya B.L. (2015). Antibacterial potential of a basic phospholipase A_2_ (VRV-PL-VIIIa) from *Daboia russelii pulchella* (Russell’s viper) venom. J. Venom. Anim. Toxins Incl. Trop. Dis..

[111] Samy R.P., Stiles B.G., Chinnathambi A., Zayed M.E., Alharbi S.A., Franco O.L., Rowan E.G., Kumar A.P., Lim L.H.K., Sethi G. (2015). Viperatoxin-II: A novel viper venom protein as an effective bactericidal Agent. FEBS Open Bio.

[112] Samy R.P., Gopalakrishnakone P., Bow H., Puspharaj P.N., Chow V.T.K. (2010). Identification and characterization of a phospholipase A_2_ from the venom of the Saw-scaled viper: Novel bactericidal and membrane damaging activities. Biochimie.

[113] Diniz-Sousa R., Caldeira C.A.S., Kayano A.M., Paloschi M.V., Pimenta D.C., Simões-Silva R., Ferreira A.S., Zanchi F.B., Matos N.B., Grabner F.P. (2018). Identification of the Molecular Determinants of the Antibacterial Activity of LmutTX, a Lys49 Phospholipase A2 Homologue Isolated from *Lachesis muta muta* Snake Venom (Linnaeus, 1766). Basic Clin. Pharmacol. Toxicol..

[114] Accary C., Mantash A., Mallem Y., Fajloun Z., Elkak A. (2015). Separation and Biological Activities of Phospholipase A2 (Mb-PLA2) from the Venom of *Montivipera bornmuelleri*, a Lebanese Viper. J. Liq. Chromatogr. Relat. Technol..

[115] Sudarshan S., Dhananjaya B.L. (2015). The Antimicrobial Activity of an Acidic Phospholipase A_2_ (NN-XIa-PLA_2_) from the Venom of *Naja naja naja* (Indian Cobra). Appl. Biochem. Biotechnol..

[116] Sudarshan S., Dhananjaya B.L. (2016). Antibacterial activity of an acidic phospholipase A2 (NN-XIb-PLA2) from the venom of *Naja naja* (Indian cobra). SpringerPlus.

[117] Adade C.M., Carvalho A.L.O., Tomaz M.A., Costa T.F.R., Godinho J.L., Melo P.A., Lima A.P.C.A., Rodrigues J.C.F., Zingali R.B., Souto-Padrón T. (2014). Crovirin, a Snake Venom Cysteine-Rich Secretory Protein (CRISP) with Promising Activity against Trypanosomes and *Leishmania*. PLoS Negl. Trop. Dis..

[118] Castillo J.C.Q., Vargas L.J., Segura C., Gutiérrez J.M., Pérez J.C.A. (2012). In Vitro Antiplasmodial Activity of Phospholipases A_2_ and a Phospholipase Homologue Isolated from the Venom of the Snake *Bothrops asper*. Toxins.

[119] Mello C.P., Lima D.B., Menezes R.R.P.P.B., Bandeira I.C.J., Tessarolo L.D., Sampaio T.L., Falcao C.B., Radis-Baptista G., Martins A.M.C. (2017). Evaluation of the antichagasic activity of batroxicidin, a cathelicidin-related antimicrobial peptide found in *Bothrops atrox* venom gland. Toxicon.

[120] Tempone A.G., Andrade H.F., Spencer P.J., Lourenço C.O., Rogero J.R., Nascimento N. (2001). *Bothrops moojeni* Venom Kills *Leishmania* spp. with Hydrogen Peroxide Generated by Its L-Amino Acid Oxidase. Biochem. Biophys. Res. Commun..

[121] Wang Y., Hong J., Liu X., Yang H., Liu R., Wu J., Wang A., Lin D., Lai R. (2008). Snake Cathelicidin from *Bungarus fasciatus* Is a Potent Peptide Antibiotics. PLoS ONE.

[122] Quintana J.C., Chacón A.M., Vargas L., Segura C., Gutiérrez J.M., Alarcón J.C. (2012). Antiplasmodial effect of the venom of *Crotalus durissus cumanensis*, crotoxin complex and Crotoxin B. Acta Trop..

[123] Yamane E.S., Bizerra F.C., Oliveira E.B., Moreira J.T., Rajabi M., Nunes G.L.C., Souza A.O., Silva I.D.C.G., Yamane T., Karpel R.L. (2013). Unraveling the antifungal activity of a South American rattlesnake toxin Crotamine. Biochimie.

[124] El Chamy Maluf S., Dal Mas C., Oliveira E.B., Melo P.M.S., Carmona A.K., Gazarini M.L., Hayashi M.A.F. (2016). Inhibition of malaria parasite *Plasmodium falciparum* development by crotamine, a cell penetrating peptide from the snake venom. Peptides.

[125] Bandeira I.C.J., Bandeira-Lima D., Mello C.P., Pereira T.P., de Menezes R.R.P.P.B., Sampaio T.L., Falcão C.B., Rádis-Baptista G., Martins A.M.C. (2018). Antichagasic effect of crotalicidin, a cathelicidin-like vipericidin, found in *Crotalus durissus terrificus* rattlesnake’s venom gland. Parasitology.

[126] Wei L., Gao J., Zhang S., Wu S., Xie Z., Ling G., Kuang Y.-Q., Yang Y., Yu H., Wang Y. (2015). Identification and Characterization of the First Cathelicidin from Sea Snakes with Potent Antimicrobial and Anti-inflammatory Activity and Special Mechanism. J. Biol. Chem..

[127] Guillaume C., Deregnaucourt C., Clavey V., Schrévela J. (2004). Anti-Plasmodium properties of group IA, IB, IIA and III secreted phospholipases A_2_ are serum-dependent. Toxicon.

[128] Wang A., Zhang F., Guo Z., Chen Y., Zhang M., Yu H., Wang Y. (2019). Characterization of a Cathelicidin from the Colubrinae Snake, *Sinonatrix annularis*. Zoolog. Sci..

[129] Nair D.G., Fry B.G., Alewood P., Kumar P.P., Kini R.M. (2007). Antimicrobial activity of omwaprin, a new member of the waprin family of snake venom proteins. Biochem. J..

[130] Torres A.M., Wong H.Y., Desai M., Moochhala S., Kuchel P.W., Kini R.M. (2003). Identification of a Novel Family of Proteins in Snake Venoms. Purification and Structural Characterization of Nawaprin from *Naja nigricollis* Snake Venom. J. Biol. Chem..

[131] Chen L.-W., Kao P.-H., Fu Y.-S., Lin S.-R., Chang L.-S. (2011). Membrane-damaging activity of Taiwan cobra cardiotoxin 3 is responsible for its bactericidal activity. Toxicon.

[132] Chen L.-W., Kao P.-H., Fu Y.-S., Hu W.-P., Chang L.-S. (2011). Bactericidal effect of *Naja nigricollis* toxin is related to its membrane-damaging activity. Peptides.

[133] Kao P.-H., Lin S.-R., Hu W.-P., Chang L.-S. (2012). *Naja naja atra* and *Naja nigricollis* cardiotoxins induce fusion of *Escherichia coli* and *Staphylococcus aureus* membrane-mimicking liposomes. Toxicon.

[134] Martin E., Ganz T., Lehrer R.I. (1995). Defensins and other endogenous peptide antibiotics of vertebrates. Leukoc. Biol..

[135] Nguyen L.T., Haney E.F., Vogel H.J. (2011). The expanding scope of antimicrobial peptide structures and their modes of action. Trends Biotechnol..

[136] Gomes V.M., Carvalho A.O., Cunha M., Keller M.N., Bloch C., Deolindo P., Alves E.W. (2005). Purification and characterization of a novel peptide with antifungal activity from *Bothrops jararaca* venom. Toxicon.

[137] Oguiura N., Boni-Mitake M., Rádis-Baptista G. (2005). New view on crotamine, a small basic polypeptide myotoxin from South American rattlesnake venom. Toxicon.

[138] Coronado M.A., Gabdulkhakov A., Georgieva D., Sankaran B., Murakami M.T., Arni R.K., Betzel C. (2013). Structure of the polypeptide crotamine from the Brazilian rattlesnake *Crotalus durissus terrificus*. Acta Cryst..

[139] Costa B.A., Sanches L., Gomide A.B., Bizerra F., Dal Mas C., Oliveira E.B., Perez K.R., Itri R., Oguiura N., Hayashi M.A.F. (2014). Interaction of the Rattlesnake Toxin Crotamine with Model Membranes. J. Phys. Chem. B.

[140] Yount N.Y., Kupferwasser D., Spisni A., Dutz S.M., Ramjan Z.H., Sharma S., Waring A.J., Yeaman M.R. (2009). Selective reciprocity in antimicrobial activity versus cytotoxicity of hBD-2 and crotamine. Proc. Natl. Acad. Sci. USA.

[141] Oguiura N., Boni-Mitake M., Affonso R., Zhang G. (2011). In vitro antibacterial and hemolytic activities of crotamine, a small basic myotoxin from rattlesnake *Crotalus durissus*. J. Antibiot. (Tokyo).

[142] Oguiura N., Corrêa P.G., Rosmino I.L., de Souza A.O., Pasqualoto K.F.M. (2022). Antimicrobial Activity of Snake β-Defensins and Derived Peptides. Toxins.

[143] Scheetz T., Bartlett J.A., Walters J.D., Schutte B.C., Casavant T.L., McCray P.B. (2002). Genomics-based approaches to gene discovery in innate immunity. Immunol. Rev..

[144] Schutte B.C., Mitros J.P., Bartlett J.A., Walters J.D., Jia H.P., Welsh M.J., Casavant T.L., McCray P.B. (2002). Discovery of five conserved β-defensin gene clusters using a computational search strategy. Proc. Natl. Acad. Sci. USA.

[145] Oliveira Y.S., Corrêa P.G., Oguiura N. (2018). Beta-defensin genes of the Colubridae snakes *Phalotris mertensi*, *Thamnodynastes hypoconia*, and *T. strigatus*. Toxicon.

[146] Corrêa P.G., Oguiura N. (2013). Phylogenetic analysis of β-defensin-like genes of *Bothrops*, *Crotalus* and *Lachesis* snakes. Toxicon.

[147] Campbell J.A., Lamar W.W. (2004). The Venomous Reptiles of the Western Hemisphere.

[148] Kaerse M., Wilson A., Stones-Havas S., Cheung M., Sturrock S., Buxton S., Cooper A., Markowitz S., Duran C., Thierer T. (2012). Geneious Basic: An integrated and extendable desktop software platform for the organization and analysis of sequence data. Bioinformatics.

[149] Rádis-Baptista G., Kubo T., Oguiura N., Svartman M., Almeida T.M.B., Batistic R.F., Oliveira E.B., Vianna-Morgante A.M., Yamane T. (2003). Structure and chromosomal localization of the gene for crotamine, a toxin from the South American rattlesnake, *Crotalus durissus terrificus*. Toxicon.

[150] Edgar R.C. (2004). MUSCLE: A multiple sequence alignment method with reduced time and space complexity. BMC Bioinform..

[151] Hall T.A. (1999). BioEdit: A user-friendly biological sequence alignment editor and analysis program for windows 95/98/NT. Nucleic Acids Symp. Ser..

[152] Rádis-Baptista G., Kubo T., Oguiura N., Silva A.R.B.P., Hayashi M.A.F., Oliveira E.B., Yamane T. (2004). Identification of crotasin, a crotamine-related gene of *Crotalus durissus terrificus*. Toxicon.

[153] Jobb G., von Haeseler A., Strimmer K. (2004). TREEFINDER: A powerful graphical analysis environment for molecular phylogenetics. BMC Evol. Biol..

[154] Strimmer K., Rambaut A. (2002). Inferring confidence sets of possibly misspecified gene trees. Proc. R. Soc. Lond. B.

[155] Zanetti M. (2004). Cathelicidins, multifunctional peptides of the innate immunity. J. Leukoc. Biol..

[156] Zhao H., Gan T.-X., Liu X.-D., Jin Y., Lee W.-H., Shen J.-H., Zhang Y. (2008). Identification and characterization of novel reptile cathelicidins from elapid snakes. Peptides.

[157] Zanetti M., Gennaro R., Romeo D. (1995). Cathelicidins: A novel protein family with a common proregion and a variable C-terminal antimicrobial domain. FEBS Lett..

[158] Kosciuczuk E.M., Lisowski P., Jarczak J., Strzałkowska N., Jozwik A., Horbanczuk J., Krzyzewski J., Zwierzchowski L., Bagnicka E. (2012). Cathelicidins: Family of antimicrobial peptides. A review. Mol. Biol. Rep..

[159] Dalla Valle L., Benato F., Paccanaro M.C., Alibardi L. (2013). Bioinformatic and molecular characterization of cathelicidin-like peptides isolated from the green lizard *Anolis carolinensis* (Reptilia: Lepidosauria: Iguanidae). Ital. J. Zool..

[160] Zanetti M., Gennaro R., Skerlavaj B., Tomasinsig L., Circo R. (2002). Cathelicidin Peptides as Candidates for a Novel Class of Antimicrobials. Curr. Pharm. Des..

[161] Du H., Samuel R.L., Massiah M.A., Gillmor S.D. (2015). The structure and behavior of the NA-CATH antimicrobial peptide with liposomes. Biochim. Biophys. Acta.

[162] Samuel R., Gillmor S. (2016). Membrane phase characteristics control NA-CATH activity. Biochim. Biophys. Acta.

[163] Azim S., McDowell D., Cartagena A., Rodriguez R., Laughlin T.F., Ahmada Z. (2016). Venom peptides cathelicidin and lycotoxin cause strong inhibition of *Escherichia coli* ATP synthase. Int. J. Biol. Macromol..

[164] de Barros E., Gonçalves R.M., Cardoso M.H., Santos N.C., Franco O.L., Cândido E.S. (2019). Snake Venom Cathelicidins as Natural Antimicrobial Peptides. Front. Pharmacol..

[165] Falcao C.B., La Torre B.G., Pérez-Peinado C., Barron A.E., Andreu D., Rádis-Baptista G. (2014). Vipericidins: A novel family of cathelicidin-related peptides from the venom gland of South American pit vipers. Amino Acids.

[166] Oliveira-Júnior N.G., Freire M., Almeida J.A., Rezende T.M.B., Franco O.L. (2018). Antimicrobial and proinflammatory effects of two vipericidins. Cytokine.

[167] Wang Y., Zhang Z., Chen L., Guang H., Li Z., Yang H., Li J., You D., Yu H., Lai R. (2011). Cathelicidin-BF, a Snake Cathelicidin-Derived Antimicrobial Peptide, Could Be an Excellent Therapeutic Agent for *Acne Vulgaris*. PLoS ONE.

[168] Hao Q., Wang H., Wang J., Dou J., Zhang M., Zhou W., Zhou C. (2013). Effective antimicrobial activity of Cbf-K16 and Cbf-A7A13 against NDM-1-carrying *Escherichia coli* by DNA binding after penetrating the cytoplasmic membrane in vitro. J. Pept. Sci..

[169] Wang J., Li B., Li Y., Dou J., Hao Q., Tian Y., Wang H., Zhou C. (2014). BF-30 effectively inhibits ciprofloxacin-resistant bacteria in vitro and in a rat model of vaginosis. Arch. Pharm. Res..

[170] Li L., Wang Q., Li H., Yuan M., Yuan M. (2014). Preparation, Characterization, In Vitro Release and Degradation of Cathelicidin-BF-30-PLGA Microspheres. PLoS ONE.

[171] Xia X., Zhang L., Wang Y. (2015). The antimicrobial peptide cathelicidin-BF could be a potential therapeutic for *Salmonella typhimurium* infection. Microbiol. Res..

[172] Liu C., Qi J., Shan B., Gao R., Gao F., Xie H., Yuan M., Liu H., Jin S., Wu F. (2018). Pretreatment with cathelicidin-BF ameliorates *Pseudomonas aeruginosa* pneumonia in mice by enhancing NETosis and the autophagy of recruited neutrophils and macrophages. Int. Immunopharmacol..

[173] Tajbakhsh M., Akhavan M.M., Fallah F., Karimi A. (2018). A Recombinant Snake Cathelicidin Derivative Peptide: Antibiofilm Properties and Expression in *Escherichia coli*. Biomolecules.

[174] Carlile S.R., Shiels J., Kerrigan L., Delaney R., Megaw J., Gilmore B.F., Weldon S., Dalton J.P., Taggart C.C. (2019). Sea snake cathelicidin (Hc-cath) exerts a protective effect in mouse models of lung inflammation and infection. Sci. Rep..

[175] Blower R.J., Barksdale S.M., van Hoek M.L. (2015). Snake Cathelicidin NA-CATH and Smaller Helical Antimicrobial Peptides Are Effective against *Burkholderia thailandensis*. PLoS Negl. Trop. Dis..

[176] Blower R.J., Popov S.G., van Hoek M.L. (2018). Cathelicidin peptide rescues *G. mellonella* infected with *B. anthracis*. Virulence.

[177] Amer L.S., Bishop B.M., van Hoek M.L. (2010). Antimicrobial and antibiofilm activity of cathelicidins and short, synthetic peptides against *Francisella*. Biochem. Biophys. Res. Commun..

[178] Latour F.A., Amer L.S., Papanstasiou E.A., Bishop B.M., van Hoek M.L. (2010). Antimicrobial activity of the *Naja atra* cathelicidin and related small peptides. Biochem. Biophys. Res. Commun..

[179] Li S.-A., Xiang Y., Wang Y.-J., Liu J., Lee W.-H., Zhang Y. (2013). Naturally Occurring Antimicrobial Peptide OH-CATH30 Selectively Regulates the Innate Immune Response to Protect against Sepsis. J. Med. Chem..

[180] Zhao F., Lan X.-Q., Du Y., Chen P.-Y., Zhao J., Zhao F., Lee W.-H., Zhang Y. (2018). King cobra peptide OH-CATH30 as a potential candidate drug through clinic drug-resistant isolates. Zool. Res..

[181] Creane S.E., Carlile S.R., Downey D., Weldon S., Dalton J.P., Taggart C.C. (2021). The Impact of Lung Proteases on Snake-Derived Antimicrobial Antimicrobial Peptides. Biomolecules.

[182] Kim D., Soundrarajan N., Lee J., Cho H.-S., Choi M., Cha S.-Y., Ahn B., Jeon H., Le M.T., Song H. (2017). Genomewide analysis of the antimicrobial peptides in *Python bivittatus* and characterization of cathelicidins with potent antimicrobial activity and low cytotoxicity. Antimicrob. Agents Chemother..

[183] Cai S., Qiao X., Feng L., Shi N., Wang H., Yang H., Guo Z., Wang M., Chen Y., Wang Y. (2018). *Python* Cathelicidin CATHPb1 Protects against Multidrug-resistant Staphylococcal Infections by Antimicrobial-Immunomodulatory Duality. J. Med. Chem..

[184] Zhang H., Xia X., Han F., Jiang Q., Rong Y., Song D., Wang Y. (2015). Cathelicidin-BF, a Novel Antimicrobial Peptide from *Bungarus fasciatus*, Attenuates Disease in a Dextran Sulfate Sodium Model of Colitis. Mol. Pharm..

[185] Yi H., Yu C., Zhang H., Song D., Jiang D., Du H., Wang Y. (2015). Cathelicidin-BF suppresses intestinal inflammation by inhibiting the nuclear factor-κB signaling pathway and enhancing the phagocytosis of immune cells via STAT-1 in weanling piglets. Int. Immunopharmacol..

[186] Zhang H., Zhang B., Zhang X., Wang X., Wu K., Guan Q. (2017). Effects of cathelicidin-derived peptide from reptiles on lipopolysaccharide-induced intestinal inflammation in weaned piglets. Vet. Immunol. Immunopathol..

[187] Li S.-A., Lee W.-H., Zhang Y. (2012). Efficacy of OH-CATH30 and Its Analogs against drug-resistant Bacteria In Vitro and in Mouse Models. Antimicrob. Agents Chemother..

[188] Nei M., Rooney A.P. (2005). Concerted and birth-and-death evolution of multigene families. Annu. Rev. Genet..

[189] Pizzolato-Cezar L.R., Okuda-Shinagawa M., Machini M.T. (2019). Combinatory Therapy Antimicrobial Peptide-Antibiotic to Minimize the Ongoing Rise of Resistance. Front. Microbiol..

[190] Mendes B., Almeida J.R., Vale N., Gomes P., Gadelha F.R., Silva S.L., Miguel D.C. (2019). Potential use of 13-mer peptides based on phospholipase and oligoarginine as leishmanicidal agents. Comp. Biochem. Physiol. C.

[191] Almeida J.R., Mendes B., Lancellotti M., Franchi G.C., Passos Ó., Ramos M.J., Fernandes P.A., Alves C., Vale N., Gomes P. (2022). Lessons from a Single Amino Acid Substitution: Anticancer and Antibacterial Properties of Two Phospholipase A2-Derived Peptides. Curr. Issues Mol. Biol..

[192] Lomonte B., Pizarro-Cerda J., Angulo Y., Gorvel J.P., Moreno E. (1999). Tyr-Trp-substituted peptide 115-129 of a Lys49 phospholipase A_2_ expresses enhanced membrane-damaging activities and reproduces its in vivo myotoxic eject. Biochim. Biophys. Acta.

[193] Santos-Filho N.A., Lorenzon E.N., Ramos M.A.S., Santos C.T., Piccoli J.P., Bauab T.M., Fusco-Almeida A.M., Cilli E.M. (2015). Synthesis and characterization of an antibacterial and non-toxic dimeric peptide derived from the C-terminal region of Bothropstoxin-I. Toxicon.

[194] Santos-Filho N.A., Fernandes R.S., Sgardioli B.F., Ramos M.A.S., Piccoli J.P., Camargo I.L.B.C., Bauab T.M., Cilli E.M. (2017). Antibacterial Activity of the Non-Cytotoxic Peptide (p-BthTX-I)2 and Its Serum Degradation Product against Multidrug-resistant Bacteria. Molecules.

[195] Peña-Carrillo M., Pinos-Tamayo E.A., Mendes B., Domínguez-Borbor C., Proaño-Bolaños C., Miguel D.C., Almeida J.R. (2021). Dissection of phospholipases A2 reveals multifaceted peptides targeting cancer cells, Leishmania and bacteria. Bioorg. Chem..

[196] Chen W., Yang B., Zhou H., Sun L., Dou J., Qian H., Huang W., Mei Y., Han J. (2011). Structure–activity relationships of a snake cathelicidin-related peptide, BF-15. Peptides.

[197] Li B., Kang W., Liu H., Wang Y., Yu C., Zhu X., Dou J., Cai H., Zhou C. (2016). The antimicrobial activity of Cbf-K16 against MRSA was enhanced by b-lactamantibiotics through cell wall non-integrity. Arch. Pharm. Res..

[198] Jin L., Bai X., Luan N., Yao H., Zhang Z., Liu W., Chen Y., Yan X., Rong M., Lai R. (2016). A Designed Tryptophan- and Lysine/Arginine-Rich Antimicrobial Peptide with Therapeutic Potential for Clinical Antibiotic-Resistant *Candida albicans* Vaginitis. J. Med. Chem..

[199] Ma L., Wang Y., Wang M., Tian Y., Kang W., Liu H., Wang H., Dou J., Zhou C. (2016). Effective antimicrobial activity of Cbf-14, derived from a cathelin-like domain, against penicillin-resistant bacteria. Biomaterials.

[200] Tajbakhsh M., Karimi A., Tohidpour A., Abbasi N., Fallah F., Akhavan M.M. (2018). The antimicrobial potential of a new derivative of cathelicidin from *Bungarus fasciatus* against methicillin-resistant *Staphylococcus aureus*. J. Microbiol..

[201] Falcao C.B., Perez-Peinado C., Torre B.G., Mayol X., Zamora-Carreras H., Jimenez M.Á., Rádis-Baptista G., Andreu D. (2015). Structural Dissection of Crotalicidin, a Rattlesnake Venom Cathelicidin, Retrieves a Fragment with Antimicrobial and Antitumor Activity. J. Med. Chem..

[202] Dal Mas C., Pinheiro D.A., Campeiro J.D., Mattei B., Oliveira V., Oliveira E.B., Miranda A., Perez K.R., Hayashi M.A.F. (2017). Biophysical and biological properties of small linear peptides derived from crotamine, a cationic antimicrobial/antitumoral toxin with cell penetrating and cargo delivery abilities. Biochim. Biophys. Acta Biomembr..

[203] Cavalcante C.S.P., Aguiar F.L.L., Fontenelle R.O.S., Menezes R.R.P.P.B., Martins A.M.C., Falcao C.B., Andreu D., Radis-Baptista G. (2018). Insights into the candidacidal mechanism of Ctn[15–34]—A carboxyl-terminal, crotalicidin-derived peptide related to cathelicidins. J. Med. Microbiol..

[204] Ponnappan N., Budagavi D.P., Chugh A. (2017). CyLoP-1: Membrane-active peptide with cell-penetrating and antimicrobial properties. Biochim. Biophys. Acta.

[205] Pérez-Peinado C., Dias S.A., Domingues M.M., Benfield A.H., Freire J.M., Rádis-Baptista G., Gaspar D., Castanho M.A.R.B., Craik D.J., Henriques S.T. (2018). Mechanisms of bacterial membrane permeabilization by crotalicidin (Ctn) and its fragment Ctn(15–34), antimicrobial peptides from rattlesnake venom. J. Biol. Chem..

[206] Priya A., Aditya A., Budagavi D.P., Chugh A. (2022). Tachyplesin and CyLoP-1 as efficient anti-mycobacterial peptides: A novel finding. Biochim. Biophys. Acta Biomembr..

[207] Almeida J.R., Mendes B., Lancellotti M., Marangoni S., Vale N., Passos Ó., Ramos M.J., Fernandes P.A., Gomes P., da Silva S.L. (2018). A novel synthetic peptide inspired on Lys49 phospholipase A2 from *Crotalus oreganus abyssus* snake venom active against multidrug-resistant clinical isolates. Eur. J. Med. Chem..

[208] Dean S.N., Bishop B.M., van Hoek M.L. (2011). Natural and synthetic cathelicidin peptides with anti-microbial and anti-biofilm activity against *Staphylococcus aureus*. BMC Microbiol..

[209] Juba M., Porter D., Dean S., Gillmor S., Bishop B. (2013). Characterization and Performance of Short Cationic Antimicrobial Peptide Isomers. Biopolymers (Pept. Sci.).

[210] Sala A., Cabassi C.S., Santospirito D., Polverini E., Flisi S., Cavirani S., Taddei S. (2018). Novel *Naja atra* cardiotoxin 1 (CTX-1) derived antimicrobial peptides with broad spectrum activity. PLoS ONE.

[211] Zhang Y., Zhao H., Yua G.-Y., Liu X.-D., Shen J.-H., Lee W.-H., Zhang Y. (2010). Structure–function relationship of king cobra cathelicidin. Peptides.

[212] De Aguiar F.L.L., Cavalcante C.S.d.P., Fontenelle R.O.S., Falcao C.B., Andreu D., Radis-Baptista G. (2019). The antiproliferative peptide Ctn[15–34] is active against multidrug-resistant yeasts *Candida albicans* and *Cryptococcus neoformans*. J. Appl. Microbiol..

[213] Perez-Peinado C., Valle J., Freire J.M., Andreu D. (2020). Tumor Cell Attack by Crotalicidin (Ctn) and Its Fragment Ctn[15–34]: Insights into Their Dual Membranolytic and Intracellular Targeting Mechanism. ACS Chem. Biol..

[214] El-Aziz T.M.A., Soares A.G., Stockand J.D. (2019). Snake Venoms in Drug Discovery: Valuable Therapeutic Tools for Life Saving. Toxins.

[215] Alam M.I., Ojha R., Alam M.A., Quasimi H., Alam O. (2019). Therapeutic potential of snake venoms as antimicrobial agents. Front. Drug Chem. Clin. Res..

[216] Boldrini-França J., Cologna C.T., Pucca M.B., Bordon K.C.F., Amorim F.G., Anjolette F.A.P., Cordeiro F.A., Wiezel G.A., Cerni F.A., Pinheiro-Junior E.L. (2017). Minor snake venom proteins: Structure, function and potential applications. Biochim. Biophys. Acta Gen. Subjects.

[217] Fry B.G., Koludarov I., Jackson T.N.W., Holford M., Terrat Y., Casewell N.R., Undheim E.A.B., Vetter I., Ali S.A., Low D.H.W., King G.F. (2015). Seeing the Woods for the Trees: Understanding Venom Evolution. Venoms to Drugs: Venom as a Source for the Development of Human Therapeutics.

[218] Rambault A. FigTree for Windows v. 1.4.4. http://tree.bio.ed.ac.uk/software/figtree/.

[219] Wheeler D.L., Barrett T., Benson D.A., Bryant S.H., Canese K., Chetvernin V., Church D.M., DiCuccio M., Edgar R., Federhen S. (2007). Database resources of the national center for biotechnology information. Nucleic Acids Res..

[220] Altschul S.F., Gish W., Miller W., Myers E.W., Lipman D.J. (1990). Basic local alignment search tool. J. Mol. Biol..

[221] Morgulis A., Coulouris G., Raytselis Y., Madden T.L., Agarwala R., Schäffer A.A. (2008). Database indexing for production MegaBLAST searches. Bioinformatics.

[222] Camacho C., Coulouris G., Avagyan V., Ma N., Papadopoulos J., Bealer K., Madden T.L. (2009). BLAST+: Architecture and applications. BMC Bioinform..

[223] R Core Team R: A Language and Environment for Statistical Computing [Internet]. Vienna, Austria, 2016. https://www.R-project.org/.

[224] Pagès H., Aboyoun P., Gentleman R., DebRoy S. Biostrings: Efficient Manipulation of Biological Strings; R Package Version 2.64.21. Bioconductor. https://rdrr.io/bioc/Biostrings/.

[225] Osorio D., Rondón-Villarreal P., Torres R. (2015). Peptides: A package for data mining of antimicrobial peptides. Small.

[226] Katoh K., Rozewicki J., Yamada K.D. (2019). MAFFT online service: Multiple sequence alignment, interactive sequence choice, and visualization. Brief. Bioinform..

