# Supplementary material for: Past, Present, and Future of Naturally Occurring Antimicrobials Related to Snake Venoms
Source: Animals (Basel). 2023 Feb 19;13(4):744. doi: 10.3390/ani13040744 (PMC9952678; doi:10.3390/ani13040744)
Supplement: Supplementary file 1 [file animals-13-00744-s001.zip › animals-2131857-supplementary.docx]

**Supplementary Materials**

1. **Material and Methods**

*1.1 β-defensin genes of Sistrurus*

1.1.1. Material

Two North American species of rattlesnake were studied, *Sistrurus catenatus* (Massasauga rattlesnake) and *S. miliarius* (Pygmy rattlesnake). Dr. H. Leslie Gibbs kindly provided DNA samples : Sca692 and Sca697 (*S. catenatus* specimens from Ohio and New York, respectively), and Smi133 and Smi136 *S. miliarius* specimens caught in Florida.

Primers: H010-cro (5´- AAG CAG TCT CAG CAT GAA GAT – 3`) and 3UTRas-cro (5´ - GGC ACT CTC AGG TCC TTG GCC AT – 3´) [146].

1.1.2. Methodology and analysis

PCR: in 25 µL of reaction: 0.2 mM dNTPs, 1.5 mM MgCl2, 0.1 µM primers, 150 ng DNA, 0.5 U Platinum Taq DNA polymerase InVitroGen; the amplification occurs at 94 °C for 4 min; followed by 30 cycles of 30 s at 94 °C, 55 °C for 30 s and 72 °C for 90 s; then 72 °C for 4 min. After purification from agarose gel using the Zymoclean Gel DNA Recovery kit, the amplicons were cloned using cloneJET PCR Cloning (Thermo Scientific) kit and sequencing using BigDye® Direct Cycle Sequencing Thermo Fisher Scientific.

Using Geneious R7® [148], the resulting sequences were concatenated, translated, and aligned. The phylogenetic tree was constructed using TreeFinder [153], with Likelihood as criterion and FLU [Empirical]:GI[Optimum]5 as model. The confidence shown in each node was analyzed using (LR-ELW [154]). The resulting tree, figure 3, was edited in FigTree v. 1.4.4 [218] for publication.

*1.2. Cathelicidins sequences*

Snake cathelicidin nucleotide sequences were searched in the literature and NCBI database [219]. Those sequences were used as queries in iterative searches on NCBI non-redundant and TSA databases using BLAST algorithms [220-222]. The 56 resulting sequences (Table S1) were also used in genome searches on the NCBI WGS database, returning 39 new partial genes from 29 contigs (Table S2). Partial or premature stop codon sequences were not used in the following analysis.

The sequences were handled using R software (Version 4.2.2) [223] and Biostrings package (Version 2.64.1) [224]; amino acid sequence and biochemical characteristics were predicted using Peptides package (Version 2.4.4) [225]. The cathelicidins were aligned (MAFFT, Version 7) [226], and the mature peptide was inferred based on the elastase-conserved cleavage site in a specific alignment region.

The tree was constructed using TreeFinder [153], with Likelihood as criterion, and MIX [Dayhoff, BLOSUM, JTT, LG, PMB, VT, WAG, mtREV, mtMam, mtArt, mtZOA, cpREV, rtREV, betHIV, witHIV, FLU] [Optimum,Empirical]: G[Optimum]:5 as model. The resulting tree, figure 4, was edited in FigTree v. 1.4.4 [218] for publication.

**Table S1. Characteristics of snake cathelicidins. The sequences were obtained from the literature or NCBI databases. The net charge was calculated using the Henderson-Hasselbalch equation and the Lehninger pKa Scale.**

| **ID GenBank** | **Species** | **Family** | **Name** | **Antibacterial**  **activity** | **Net charge**  **at pH 7** | **Reference** |
| --- | --- | --- | --- | --- | --- | --- |
| ACF21000 | *Naja atra* | Elapidae | Na_CRAMP | Active | +15 | [156] |
| KAG8148195 | *Naja naja* | Elapidae |  | Not tested | +4 |  |
| ACF21002 | *Ophiophagus hannah* | Elapidae | Oh-Cath | Active | +15 | [156] |
| ACI22652 | *Bungarus fasciatus* | Elapidae | cathelicidin-BF | Active | +14 | [121] |
| AGS36143 | *Pseudonaja textilis* | Elapidae | Pt_CRAMP2 | Active | +16 | [166] |
| AGS36144 | *Pseudonaja textilis* | Elapidae | Pt_CRAMP1 | Active | +13 | [166] |
| XP026580595 | *Pseudonaja textilis* | Elapidae | Pt_CRAMP2 | Not tested | +16 |  |
| AKJ54480 | *Hydrophis cyanocinctus* | Elapidae | Hc-CATH | Active | +15 | [126] |
| QBZ68899 | *Trimerodytes annularis* | Colubridae | SA-CATH | Active | +15 | [128] |
| XP013912467 | *Thamnophis sirtalis* | Colubridae |  | Not tested | +16 |  |
| XP032093162 | *Thamnophis elegans* | Colubridae |  | Not tested | +16 |  |
| XP034262502 | *Pantherophis guttatus* | Colubridae |  | Not tested | ND |  |
| AGS36136 | *Crotalus durissus cascavella* | Viperidae |  | Not tested | +26 |  |
| AGS36138 | *Crotalus durissus terrificus* | Viperidae | crotalicidin 3 | Active | +15 | [166] |
| AGS36139 | *Crotalus durissus cascavella* | Viperidae | crotalicidin 1 | Active | +15 | * |
| AGS36142 | *Lachesis muta rhombeata* | Viperidae | lachescidin B7 | Active | +15 | [166] |
| AGS36137 | *Crotalus durissus cascavella* | Viperidae | crotalicidin 2 E2 | Active | +15 | * |
| AGS36140 | *Bothrops atrox* | Viperidae | batroxcidin 1 | Active | +15 | [166] |
| AGS36141 | *Bothrops lutzi* | Viperidae | lutzscidin B | Active | +15 | [166] |
| KAG8148197 | *Naja naja* | Elapidae |  | Not tested | ND |  |
| XP032093537 | *Thamnophis elegans* | Colubridae |  | Not tested | +12 |  |
| XP034260395 | *Pantherophis guttatus* | Colubridae |  | Not tested | +11 |  |
| KAG8148193 | *Naja naja* | Elapidae |  | Not tested | ND |  |
| XP013928483 | *Thamnophis sirtalis* | Colubridae |  | Not tested | +11 |  |
| XP034262044 | *Pantherophis guttatus* | Colubridae |  | Not tested | ND |  |
| XP026550105 | *Notechis scutatus* | Elapidae |  | Not tested | ND |  |
| XP032073060 | *Thamnophis elegans* | Colubridae |  | Not tested | +11 |  |
| XP032090770 | *Thamnophis elegans* | Colubridae |  | Not tested | +18 |  |
| XP032090771 | *Thamnophis elegans* | Colubridae |  | Not tested | +23 |  |
| XP039224740 | *Crotalus tigris* | Viperidae |  | Not tested | +7 |  |
| XP039224747 | *Crotalus tigris* | Viperidae |  | Not tested | +14 |  |
| XP039224748 | *Crotalus tigris* | Viperidae |  | Not tested | +14 |  |
| XP007442672 | *Python bivittatus* | Pythonidae | CATHPb2 | Active | +9 | [184] |
| XP015669791 | *Protobothrops mucrosquamatus* | Viperidae |  | Not tested | +12 |  |
| XP039224688 | *Crotalus tigris* | Viperidae |  | Not tested | +12 |  |
| XP034260397 | *Pantherophis guttatus* | Colubridae |  | Not tested | +12 |  |
| XP007442673 | *Python bivittatus* | Pythonidae | CATHPb5 | No active | -4 | [184] |
| XP039224690 | *Crotalus tigris* | Viperidae |  | Not tested | +8 |  |
| XP039224691 | *Crotalus tigris* | Viperidae |  | Not tested | +7 |  |
| XP007445036 | *Python bivittatus* | Pythonidae | CATHPb4 | Active | +9 | [184] |
| XP025032035 | *Python bivittatus* | Pythonidae | CATHPb6 | No active | +6 | [184] |
| XP007443270 | *Python bivittatus* | Pythonidae | CATHPb1 | Active | +13 | [184] |
| XP007445262 | *Python bivittatus* | Pythonidae | CATHPb3 | No active | +6 | [184] |
| XP013917356 | *Thamnophis sirtalis* | Colubridae |  | Not tested | ND |  |
| XP034260400 | *Pantherophis guttatus* | Colubridae |  | Not tested | +6 |  |
| XP015682911 | *Protobothrops mucrosquamatus* | Viperidae |  | Not tested | +3 |  |
| XP029142808 | *Protobothrops mucrosquamatus* | Viperidae |  | Not tested | +3 |  |
| XP039224689 | *Crotalus tigris* | Viperidae |  | Not tested | +3 |  |
| KAG8148196 | *Naja naja* | Elapidae |  | Not tested | +13 |  |
| XP026545397 | *Notechis scutatus* | Elapidae |  | Not tested | ND |  |
| XP026579196 | *Pseudonaja textilis* | Elapidae |  | Not tested | +5 |  |
| XP013912465 | *Thamnophis sirtalis* | Colubridae |  | Not tested | +3 |  |
| XP032090774 | *Thamnophis elegans* | Colubridae |  | Not tested | +13 |  |
| XP034260401 | *Pantherophis guttatus* | Colubridae |  | Not tested | ND |  |
| XP039224720 | *Crotalus tigris* | Viperidae |  | Not tested | +11 |  |
| XP039224721 | *Crotalus tigris* | Viperidae |  | Not tested | +2 |  |

* Comment in NCBI Protein, consulted in 08 July 2022. ND = No data.

**Table S2.** **Snake cathelicidins genome position. The contigs containing cathelicidin genes were identified through recursive BLAST searches of the WGS NCBI database, and the approximate positions of the genes were recorded. The sequences represented in this table are all partial and have not been previously reported in the literature.**

| **Contig ID GenBank** | **Organism** | **Number of Genes** | **Start** | **End** |
| --- | --- | --- | --- | --- |
| AZIM01000106.1 | *Ophiophagus hannah* | 2 | 280000 | 310000 |
| BHEV01045428.1 | *Emydocephalus ijimae* | 1 | 9200 | 15000 |
| BHFS01011480.1 | *Hydrophis melanocephalus* | 1 | 20000 | 31000 |
| BHFT01017477.1 | *Laticauda laticaudata* | 1 | 6000 | 20000 |
| BHFT01023880.1 | *Laticauda laticaudata* | 1 | 50000 | 44000 |
| BLBF01000310.1 | *Laticauda colubrina* | 2 | 410000 | 380000 |
| JAAZTL010000005.1 | *Hydrophis cyanocinctus* | 2 | 86760000 | 86828000 |
| JABAHG010000005.1 | *Hydrophis curtus* | 2 | 167355000 | 167385000 |
| JACCQE010000009.1 | *Naja naja* | 1 | 2356000 | 2400000 |
| JAGDQI010000004.1 | *Bungarus multicinctus* | 1 | 2977000 | 2974000 |
| JAHUAB010013451.1 | *Pituophis catenifer pumilus* | 1 | 900 | 3000 |
| JAHWGE010025090.1 | *Chrysopelea ornata* | 1 | 100 | 2000 |
| JAHWGE010633351.1 | *Chrysopelea ornata* | 1 | 4000 | 1000 |
| JAKOOJ010000052.1 | *Arizona elegans* | 2 | 2185582 | 2220000 |
| JTLQ02000251.1 | *Pantherophis guttatus* | 1 | 191000 | 196000 |
| JTLQ02014076.1 | *Pantherophis guttatus* | 1 | 209000 | 206000 |
| LFLD01074338.1 | *Thamnophis sirtalis* | 1 | 1000 | 6000 |
| QLTV01004414.1 | *Thermophis baileyi* | 1 | 40800 | 52000 |
| SOZL01001814.1 | *Naja naja* | 1 | 44000 | 32000 |
